# Six new species and one new subspecies of noctuid moths from western United States of America and Mexico (Lepidoptera, Noctuidae)

**DOI:** 10.3897/zookeys.788.26282

**Published:** 2018-10-08

**Authors:** Lars G. Crabo, Paul C. Hammond, Tomas Mustelin, David L. Wikle

**Affiliations:** 1 724 14th Street, Bellingham, Washington 98225, USA; 2 Adjunct Faculty, Department of Entomology, Washington State University, Pullman, Washington, USA; 3 Research Associate, Department of Integrative Biology, Oregon State University, Corvallis, Oregon, USA; 4 Division of Rheumatology, School of Medicine, University of Washington, 750 Republican Street, Seattle, Washington 98109, USA; 5 Research associate, San Diego Natural History Museum, San Diego, California, USA; 6 1021 Roanoke Road, San Marino, California 91108, USA; 7 Field Associate, Los Angeles County Museum of Natural History, Los Angeles, California

**Keywords:** Amphipyrinae, Brickellia, Cuculliinae, DNA barcode, Eriopygini, Noctuinae, Oncocnemidinae, Xylenini

## Abstract

Six new species and one new subspecies of Noctuidae are described from western United States of America and Baja California, Mexico: *Dolocuculliapoolei* Crabo & Hammond, **sp. n.** (Cuculliinae), *Plagiomimicusyakama* Crabo & Wikle, **sp. n.**, *Plagiomimicusyakamamojave* Wikle & Crabo, **ssp. n.**, *Plagiomimicusincomitatus* Mustelin, **sp. n.** (Amphipyrinae), *Sympistisferrirena* Crabo, **sp. n.** (Oncocnemidinae), *Aseptisharpi* Crabo & Mustelin, **sp. n.**, and *Hypotrixlactomellis* Wikle & Crabo, **sp. n.** (Noctuinae). The adults and genitalia of these species are described, illustrated, and compared to similar related moths. The larvae of the *Plagiomimicustepperi* species group, unknown previously, are reported to feed on several species of *Brickellia* Ell. (Asteraceae). The early stages of *Plagiomimicusyakamamojave* are described and late instars are illustrated.

## Introduction

Undescribed species of moths are still found with regularity in North America north of Mexico. This is especially true in the American West. As part of the continuing effort to document the moth fauna of the region we describe five new species and one new subspecies from four different noctuid subfamilies from the United States, mostly from the Southwest and Pacific Coast states. An additional Mexican species of *Plagiomimicus* Grote (Amphipyrinae) belonging to the same species-group as other new taxa in this paper is also described.

The species named in this paper are not related closely, but all belong to genera that have been revised since the mid-1990s and are not in need of sweeping changes. It is reasonable, therefore, to combine the descriptions into a single work in the “Contributions to the Systematics of New World Macro-moths” series. In order to provide appropriate context, this paper is organized phylogenetically and the taxon descriptions are preceded by short introductions to the pertinent genera.

In addition to the new species descriptions, early stages of the *Plagiomimicustepperi* species group, to which the new *Plagiomimicus* taxa in this paper belong, are reported for the first time. Several species in this species-group have been reared by DLW and are described as they pertain to the new taxa.

## Materials and methods

Wing pattern and genitalia structure terminology follow Lafontaine (2004). A dark ovoid spot between the postmedial and subterminal lines on the forewing costa of some Stiriini is herein referred to as the “subapical spot.” In *Aseptis* McDunnough, a pale marking straddling the postmedial line distal to the reniform stigma is referred to as the postreniform patch (Mustelin and Crabo 2015).

Forewing lengths are measured to the nearest half-millimeter from base to apex, excluding the fringe.

Genitalia were prepared using standard methods (Hardwick 1950, Lafontaine 2004). Detached abdomens were macerated in hot 10% potassium hydroxide for 20–40 minutes. Dissection was performed initially in water, or a 70 : 30 water : ethanol mixture, followed by hardening in isopropyl alcohol. Male vesicas and female bursae were inflated. Preparations were stained with orcein (Sigma Chemical Company, St. Louis, Missouri) and mounted in Euparal (Bioquip Products Inc., Rancho Dominguez, California) on glass slides. Genitalia preparations of male *Sympistis* and *Plagiomimicusincomitatus* are from USNM and are stained differently with an unknown dye.

The 658 base pair DNA “barcode region” of the mitochondrial cytochrome *c* oxidase subunit 1 (CO1) (barcode) was used to assess molecular variation. Legs from dried specimens submitted to the Barcodes of Life Data Systems (BOLD) at the University of Guelph (Ontario, Canada) were analyzed by standard DNA extraction, amplification, and sequencing protocols (Hebert et al. 2003). Barcode sequences were compared to pre-existing material at BOLD using the Kimura-2-Parameter distance model as implemented on the Barcodes of Life Data Systems website (http://www.barcodinglife.org). The seven-unit BOLD Barcode Index Number (BIN) (Ratnasingham and Hebert 2013) is given in parentheses when known.

Distribution maps were made using SimpleMappr (http://simplemappr.net).

Repository abbreviations:

**NHML** Natural History Museum (formerly, British Museum of Natural History), London, England

**CH** Chuck Harp Collection, Littleton, Colorado, USA

**CNC** Canadian National Collection of Insects, Arachnids, and Nematodes, Ottawa, Ontario, Canada

**CSUC** Colorado State University Collection, Fort Collins, Colorado, USA

**DLW** Dave Wikle Collection, San Marino, California, USA

**DNHC** Denver Museum of Nature and Science, Denver, Colorado, USA

**ER** Evan Rand Collection, Phoenix, Arizona, USA

**JS** Jon Shepard Collection, Corvallis, Oregon, USA

**LGC** Lars Crabo Collection, Bellingham, Washington, USA

**MLR** Mike Raschko Collection, Wilsonville, Oregon, USA

**MSU** Albert J. Cook Arthropod Research Collection, Michigan State University, East Lansing, Michigan, USA

**OSAC** Oregon State Arthropod Collection, Corvallis, Oregon, USA

**SDMC** San Diego Natural History Museum, San Diego, California, USA

**TM** Tomas Mustelin Collection, Seattle, Washington, USA

**USNM** Smithsonian Institution (formerly United States National Museum), Washington, DC, USA

## Systematics

### Noctuidae Latreille, 1809

#### Cuculliinae Herrich-Schäffer, [1850]

##### *Dolocucullia* Poole, 1995

Poole (1995) described *Dolocucullia* for two species from western United States and Mexico, *Dolocuculliadentilinea* (Smith, 1899) and *Dolocuculliaminor* (Barnes & McDunnough, 1913), noting that there are additional species in Central and South America. The genus resembles *Cucullia* Schrank in general appearance and structure. Males differ from *Cucullia* in that the cornuti on the vesica are globular instead of spikelike. In *Dolocucullia* females, there is a sclerite between the ovipositor lobes, lacking in *Cucullia*, and the ductus seminalis joins the corpus bursae at the posterior rather than the anterior end (op. cit.).

A relatively common *Dolocucullia* in the coastal Pacific Northwest region has until now been referred to as *D.dentilinea*. The recent discovery of *D.dentilinea* in eastern Oregon and Idaho led PCH to wonder if the disjunct coastal and inland populations could be different species. Independently, JD Lafontaine alerted LGC to large barcode differences between *Dolocucullia*’s from the Rocky Mountain and West Coast regions. Consistent differences in structure and habitus confirm that these populations are different species. The West Coast species is described herein.

A key to the three species of *Dolocucullia* found in the United States is presented below. *Dolocuculliaminor* and *D.dentilinea* species accounts are presented in Poole (1995).

###### Key to *Dolocucullia* adults of North America north of Mexico

**Table:** 

1	Dorsal hindwing ground color pure white, dark markings limited to terminal line and weak distal suffusion in females; SE Arizona to W Texas	*** D. minor ***
–	Dorsal hindwing grayish off-white with broad gray marginal band; western North America, including Arizona and New Mexico	**2**
2	Antemedial and postmedial lines not fused across medial area; male mid-sacculus width / distal valve width < 2; female corpus bursae length / width > 3; Rocky Mountain region as far west as eastern Oregon and Arizona	*** D. dentilinea ***
–	Antemedial and postmedial lines fused across medial area; male mid-sacculus width / distal valve width > 2; female corpus bursae length / width < 3; West Coast, as far east as the Sierra Nevada in California and the Cascade Range in Oregon	*** D. poolei ***

###### Dolocucullia poolei

Taxon classificationAnimaliaLepidopteraNoctuidae

Crabo & Hammond sp. n.

http://zoobank.org/08E66C35-FB8B-4614-9D5C-3C6AF76C3D00

**Figures 1–6.**
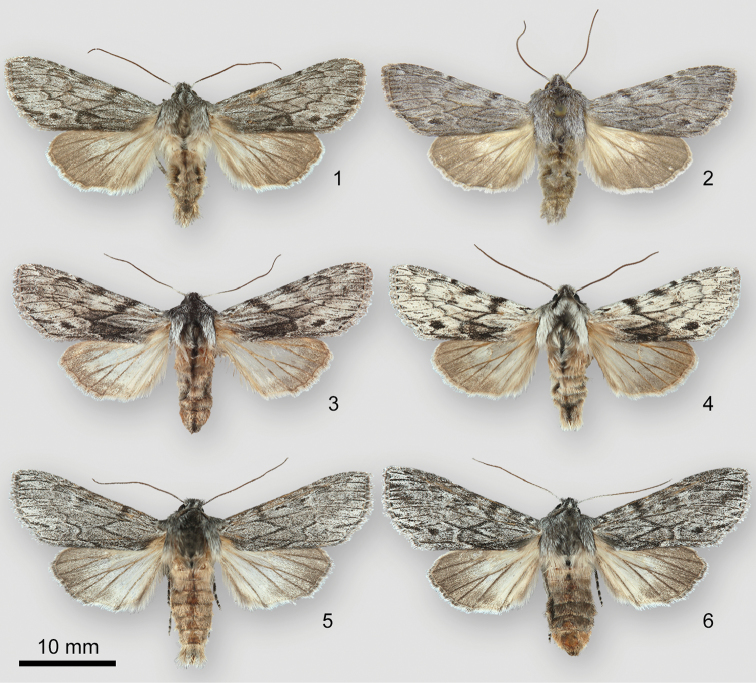
*Dolocucullia* adults. **1***D.poolei*, male, USA, Oregon, Clatsop County, Elsie, Gronnel Road **2***D.poolei*, holotype male, USA, Oregon, Marion County, Salem **3***D.poolei*, female, USA, California, Mono County, Tioga Pass, Saddlebag Lake to Warren Fork **4***D.poolei*, male, same locality as last specimen **5***D.dentilinea*, male, USA, Idaho, Franklin County, Willow Flat Campground **6***D.dentilinea*, female, same locality as last specimen.

**Figures 7–10.**
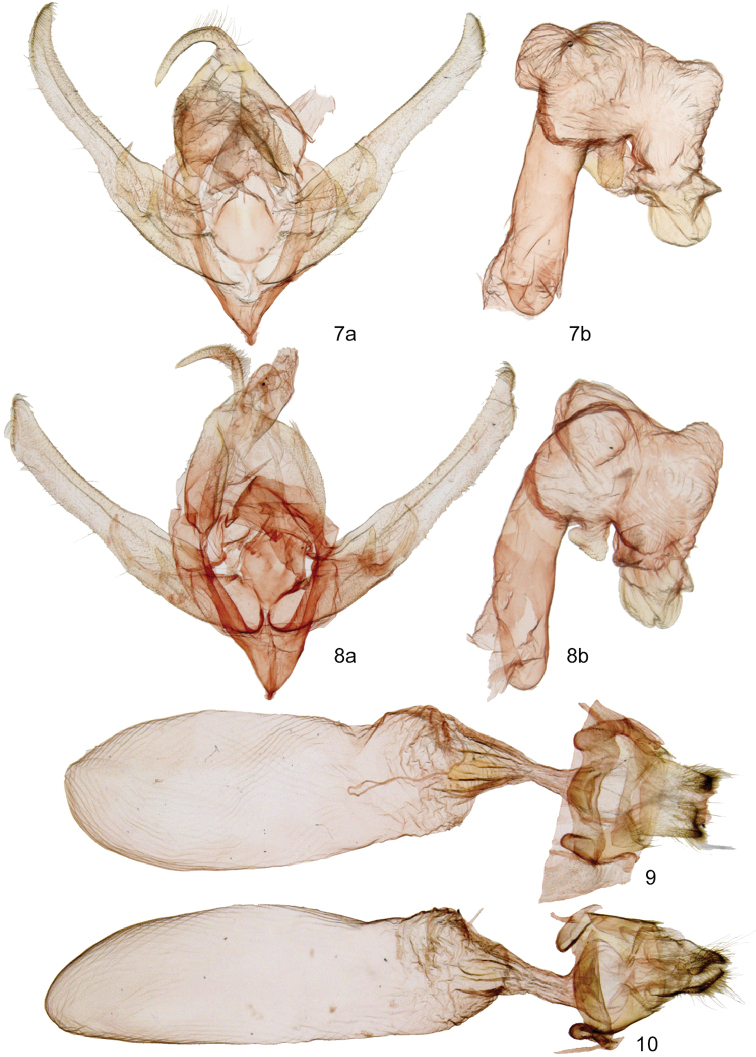
*Dolocucullia* genitalia. **7***D.poolei*, male **a** valves **b** phallus with everted vesica **8***D.dentilinea*, male **a** valves **b** phallus with everted vesica **9***D.poolei*, female **10***D.dentilinea*, female.

Figs 1–4 , 7 , 9 , 48

####### Type locality.

USA, Oregon, Marion County, Salem.

####### Type material.

**Holotype, male.** USA, Oregon, [Marion County], Salem, Blk Lt Trap, 20 VII 1959, Ken Goeden. CNC. **Paratypes.** 18 males, 18 females. **USA**: **California**: Alameda County: Oakland, 22 VI [19]08, J. R. Pilate / ex. Coll. Wolley-Dod / (*Xylina*) *dentilinea* Sm. A little darker [illegible] than the female type (xd I.10. Dod) (1 m); Marin County: Mill Valley, 20 IV [19]50 / H. B. Leech Collector / Genitalia CNC slide # 17409 female (1 f); Mendocino County: Albion, 14 VIII [no year], J. Sinclair / 15–7 / ex Coll. Wolley-Dod (1 f); Laytonville, 14 VII [no year], J. Sinclair / 15–7 / *C.dentilinea* ex. Coll. Wolley-Dod (1 m); Monterey County: Carmel, 10 VI [19]36, E. C. Johnston (1 f); High Meadow, Carmel, 36.562°N, 121.907°W, 19 IV 1991, F. P. Sala (1 m); San Diego County: S rim of Peñasquitos Canyon, 32°55.4676'N, 117°10.209'W, 5 V 2000, T. Mustelin (1 m); Sonoma County: Petaluma, 8 VI [19]39, Wm. R. Bauer Collector (1 f); Riverside County: 7.8 km N Aguanga, Wilson Vly Pres., 33.511°, -116.879°, 701 m, 10 III 2016, UV lt., C. Schmidt, D. Wikle CNC538380 (1 m); Same label data as last / CNC538381 (1 m); **Oregon**: Benton County: Marys Peak, 4021 ft. [1226 m], 7 VII 1991, [no collector] / OSAC_0000164672 (1 f); Corvallis, 225 ft. [69 m], 28 VI 1995, [no collector] / OSAC_0000164718 (1 f); Huntington Drive 4 mi. [6.4 km] N Corvallis, 18 VII 2007, J.C. Miller / OSAC_0000133697 (1 f); Philomath, Blakesley Creek, 300 m, 29 VI 2000, AVZ Brower leg. / OSAC_0000133713 (1 f); same locality & collector 1 VII 2000 / OSAC_0000133690 (1 f); same locality & collector, 24 VII 2001 / OSAC_0000133715 (1 f); same locality & collector, 25 VII 2001 / OSAC_0000133680 (1 f); Philomath, Woods Creek, 100 m, 14 VII 1999, AVZ Brower leg. / OSAC_0000133709 (1 f); Clatsop County: Gronnel Rd., Elsie, 24 V 1988, [no collector] (1 m); vic. Gronnel Rd., 2 mi. [3.2 km] E. Elsie, 4 VIII [19]63, Leg. S. G. Jewett, Jr. (1 f); same locality and collector, 9 VIII [19]63 (1 f); Lane County: nr. Triangle Lake, 692 ft. [211 m], 15 VII 1997, [no collector] / OSAC_0000164674 (1 f); Lincoln County: Siletz 25 km NE, 484 m, 24 VI 2012, [no collector] / OSAC_0000445287 & OSAC_ 0000445288 (2 m); same locality, 19 VII 2012 / OSAC_0000445292 (1 m); same locality, 23 VI 2014. OSU / OSAC_0000448088 (1 m); same locality, 22 VII 2014. OSU. / OSAC_0000448107 (1 f); Nelscott, 9 VI [no year], 0 ft [0 m], C.W. Nelson / OSAC_0000164651 (1 m); Linn County: Hwy. 20, Santiam Pass, 29 NI 1995 / OSAC_0000164662 (1 m); Marion County: same locality, date, and collector as holotype / Genitalia CNC slide # 17398 male (1 m); Polk County: Luckiamute R., 349 m, 19 VII 2012, [no collector] / OSAC_0000445266 (1 m); Tillamook County: Coast Range, Tillamook R., 28 VI 2012, [no collector] / OSAC_0000445111 (1 m); Coast Range, Wilson R., 682 m, 26 VI 2012, [no collector] / OSAC_0000445118 (1 m); Coast Range, Trask R., 934 m, 30 VII 2012, [no collector] / OSAC_0000445367 (1 f); Tillamook 30 km NE, 657 m, 18 VII 2012, [no collector] / OSAC_0000445325 (1 m). CNC, JS, LGC, OSAC.

####### Differential diagnosis.

*Dolocuculliapoolei* sp. n. (Figs 1–4) is similar to *Dolocuculliadentilinea* (Figs 5, 6), both in habitus and structure. Since they are allopatric, specimens can be assigned to species by locality: those from California, Oregon west of the Cascade crest, and Washington are *D.poolei* and those from east of California and central Oregon are *D.dentilinea*. Both are distinguished easily from *Dolocuculliaminor*, found in Texas, by hindwing color, white in *D.minor* and darker gray in the others.

*Dolocuculliapoolei* and *D.dentilinea* are similar, but can usually be identified without dissection. The forewing lines of *D.poolei* are less distinct than those of *D.dentilinea*, appearing out of focus, whereas those of *D.dentilinea* are thin and crisp. The antemedial and postmedial lines of *D.poolei* are strongly zigzagged, joining once or twice across the medial area. Those of *D.dentilinea* are usually separate. The black spot near the tornus, conspicuous in *D.poolei*, is absent or small and faint in *D.dentilinea*, especially in males. The hindwing base is darker in *D.poolei* than in *D.dentilinea*, gray with a luteous cast in the former and nearly white in the latter. A typical *D.dentilinea* is shown as Figure 5; Figure 6 demonstrates an uncommonly-patterned female with fused lines and a tornal spot.

Structurally, males of *D.poolei* (Figure 7) and *D.dentilinea* (Figure 8) are similar. Both have a two-pronged clasper with medial and lateral spikes, clasper single in *D.minor*. Compared to *D.dentilinea*, *D.poolei* has wider valve base, more cephalad orientation of the base of the lateral ampulla spike, a shorter medial ampulla spike, and a smaller cucullus with fewer coronal setae. The width of the mid-sacculus divided by the width of the distal valve is greater than two in *D.poolei* (2.2–2.3), less than two in *D.dentilinea* (1.6–1.8). The vesicas are similar, but the left-sided diverticulum of the vesica is larger and the apical “sclerotized globule” of *D.poolei* is smaller than the corresponding structures of *D.dentilinea*.

The female corpus bursae of *D.poolei* (Figure 9) is slightly wider and shorter than that of *D.dentilinea* (Figure 10). The ratio of length to width is less than three in *D.poolei*, greater than that in *D.dentilinea*. Posterior segment A8 has convex lateral margins on each side of the ostium in *D.poolei*, forming a shallow “M,” but is nearly straight in *D.dentilinea*.

The barcodes of *D.dentilinea* (BOLD:AAF5239) and *D.poolei* (BOLD:AAF5240) differ by 8.0 %. Intraspecific variation is 1.3 % in *D.dentilinea* (*n* = 7; Arizona, Colorado, Idaho, New Mexico) and 1.9 % in *D.poolei* (*n* = 11; California, Washington).

####### Description.

**Adult.***Head.* Antenna of both sexes filiform, pubescent, single short cilia on anterior and posterior sides, dorsal scales sparse, very small, light gray. Scape long with anterior loose tuft, scales white, dark gray. Eye normal, surrounded densely by thin hair-like dark scales. Labial palpus scales dense, long, mixed strap-like white and hair-like dark gray, darkest laterally, longest anteriorly; apical segment short, scales mostly white. Haustellum normal. Frons scales dense, strap-like, mixed light and dark gray, forming median ridge. Dorsal head scales long, white, dark gray, lightest at antenna base and vertex, loose anterior tuft between antennae. *Thorax.* Dorsum, including patagium and tegula, scales long, narrow, hair-like, or apically forked, mixed white, light gray, and dark gray; appearing medium gray, darkest centrally; collar broadly striped, crested. Venter scales long, hair-like, light gray. *Legs*: Tibiae lacking spines; tarsal segments except terminal segment with three rows of spine-like setae. *Wings*: Forewing: Length 15.0–16.5 mm (males), 15.0–17.0 mm (females); elongate, length 2 × width, not strongly pointed, outer margin smoothly convex, strongest near anal angle; dorsal scales elongate, rounded, mixed white, light gray, dark gray; appearing slightly mottled medium gray, medial area posterior to cell darker; Sierra Nevada population lighter gray with contrasting dark areas; veins black, thin, Cu thick across medial area; basal line absent; antemedial line black, costa to Cu thick, indistinct, angled slightly distad, Cu to 1A+2A toothed across medial area to postmedial line, segment near posterior margin less strongly so, reaching postmedial line in some specimens; medial line black, anterior segment similar to anterior antemedial line, then obsolete; postmedial line black, thinner than antemedial line, followed by light gray in most specimens, scalloped, costal origin anterior to reniform stigma, broadly convex around stigma, then nearly straight to mid-posterior margin; subterminal line pale gray or absent, irregular, preceded by indistinct dark gray shade anteriorly and prominent ill-defined dark gray to black spot near tornus; terminal line of intervening small black spots, darkest and longest crossing CuA2 toward tornal spot; fringe light to medium gray, luteous; claviform stigma absent; orbicular stigma absent or small dark gray streak with pale halo; reniform stigma faint to moderately prominent dark gray lunule or smudge. Hindwing: ground light gray, slightly luteous, distal half darker gray; veins dark; discal spot very faint; fringe off-white, base striped yellow, gray. *Abdomen* – Coremata absent. *Male genitalia*: Uncus base and mid-section oval, distal cylindrical, arced evenly, apex with short slightly downturned spine. Juxta heart shaped, broad, height = width. Valve long, gracile, width at mid-sacculus 0.2 × length, tapered slightly to ampulla of clasper, distal half narrower, even, width 0.1 × valve length; cucullus slightly wider than adjacent valve, pointed bluntly, corona simple, ~ 20 claw-like setae; sacculus 0.4 × valve length and 0.33 × width; clasper base short, ampulla bifid with spike-like medial and lateral prongs extending dorsad from base near ventral margin, right longer than left; medial component triangular, directed dorsad and 20° distad, right process 0.6 × valve width, left process slightly shorter; lateral process length 1 × valve width, base directed distad and 30° cephalad from ventral mid-valve, apex upturned to just dorsal to valve edge; digitus absent. Phallus tubular, length 3.2 × width, bent slightly ventrad. Vesica bulbous, ~ 1.25 × as long and ~ 2.5 × as wide as phallus, bent 90° rightward and slightly ventrad at base and 90° rostrad near apex to end to right of phallus; medium-sized subbasal domed diverticulum directed ventrad and leftward from left side; larger subbasal conical diverticulum directed dorsad from dorsal surface bearing small rugose sessile transversely-oriented apical cornutus; two additional globular cornuti: moderate-size foot-shape directed rostrad from mid-vesica between distal phallus and large, complex, irregular, with flat base and perpendicular rhomboid apex from anterodorsal apex. *Female genitalia*: Papilla analis pad-like, blunt, lobes joined by dorsal sclerite, covered sparsely with hair-like setae, longest at base. Segment A8 length 1.8 × width, broad invagination across posterior margin, each side of ostium bursae slightly convex caudad. Posterior apophysis 0.8 × segment A8 length; anterior apophysis 0.8 × posterior apophysis. Ostium bursae funnel shaped. Ductus bursae length 1.25 × segment A8 length, tapering evenly from ostium to near mid-point, widening gradually to broad attachment to corpus bursae. Corpus bursae length 6.3 × ductus length, membranous, ovate, length 3.3 × width, blunt posterior end expanded slightly ventrad and leftward, ductus seminalis at apex.

####### Geographic variation.

Coastal *D.poolei* (Figs 1, 2) are uniform slightly bluish gray. Sierra Nevada populations (Figs 3, 4) are mottled whitish gray with contrasting dark markings. The barcodes of these populations are not significantly different. Specimens from the Sierra Nevada are excluded from the type series because of these differences.

####### Etymology.

The name honors Robert Poole for his work on the Noctuidae of North America. He laid the groundwork for the *Dolocucullia* and *Plagiomimicus* descriptions in this paper.

####### Distribution and ecology.

*Dolocuculliapoolei* occurs near the Pacific Coast from southern California to the tip of the Olympic Peninsula, Washington (Figure 48). It is most common in the California and Oregon Coast Ranges, with records as far inland as the Oregon Cascade Range. It is restricted to the immediate coast in Washington. An apparently disjunct population occurs in the Sierra Nevada, California.

*Dolocuculliapoolei* occurs in a variety of habitats, including conifer forest, coastal chaparral, and dry mountain chaparral. It has a long flight season, from as early as March in southern California to as late as August in the Pacific Northwest. The Sierra Nevada population flies at high elevation near timberline during mid- to late summer. *Dolocuculliapoolei* is unusual for a noctuid in that females are collected at lights as often as males. The early stages are unknown.

####### Discussion.

Draudt (in Seitz 1924) named Cuculliadentilineaformmexicanus Draudt, 1924 and *Cuculliaemungens* Draudt, 1924. The type localities for both is “Mexico.” Form mexicanus describes specimens with “rusty yellow spots” found amongst more typical Mexican specimens of *D.dentilinea* that had been compared by Draudt to material from Arizona and Colorado. The Mexican Draudt types are destroyed according to Poole (1995). Illustrations of both taxa have been examined in Seitz (1924) to ensure that neither name applies to the species named *D.poolei* herein.

The barcode difference of 8 % between *D.poolei* and *D.dentilinea* is large for congeneric noctuids, and somewhat surprising given the similarity of the adult moths.

#### Amphipyrinae Guenée, 1837

##### Stiriini Grote, 1882

###### Subtribe Stiriina Grote, 1882

The Stiriini of North America north of Mexico were revised by Poole (1995). These generally attractive moths are found mostly in the southwest United States and Mexico. They have a short tubular male vesica with basal and mesial patches of spine-like cornuti, a frontal process with a raised outer ring and central cone, a reduced scale-like larval spinneret, and adaptations to desert habitats including a distal foretibial claw (op. cit.). The known larvae feed on flowers of Asteraceae. The female ovipositor lobes are sclerotized strongly and pointed, likely for inserting eggs into buds or flowers.

Many of the genera in the tribe are similar. Females of *Plagiomimicus* Grote, the largest genus in the tribe, have modified ovipositor lobes and lack clear areas on sternite A8 or an invagination of the ostium bursae found in related genera. Males typically have a simple valve with a weak setal corona, a rod-like basal process of the sacculus, and a short ampulla of the clasper from the ventral distal valve (Poole 1995).

*Plagiomimicustepperi* Morrison, 1875 and related species form a species-group distinguished by an elongate central process of the frontal tubercle and loss of the corona of the valve and the basal patch of cornuti of the vesica of the male genitalia (Poole 1995). Members of the species-group are small (forewing length 9–15 mm) and have smooth pale grayish green to ochre-yellow forewings with even white transverse lines and faint to dark subapical spots. Poole recognized two similar species in this group from the United States, *P.tepperi* and *Plagiomimicusmimica* Poole, 1995, and mentioned an undescribed central Mexican species.

Poole (1995) considered *P.tepperi*, type locality Texas, to be a widespread and geographically variable species. Under his broad concept of the species, the westernmost populations differ from topotypical populations in color and pattern, either grayer (Pacific Northwest) or paler (Great Basin and California). Poole discussed and illustrated the Washington State population, noting that it differs from nominate *P.tepperi* in habitus and the genitalia of both sexes. Despite this, he maintained this population as *P.tepperi*, arguing that *P.tepperi* is a variable moth and that these differences fall within its range of variation.

More recently, the barcodes of the Washington and pale southern populations were found to be nearly identical, and these differ from the barcode of southeastern Arizona *P.tepperi* by a magnitude similar to the difference between those of *P.tepperi* and *P.mimica*. This provides new evidence that these western *Plagiomimicus* populations are a different species than *P.tepperi*. Herein we confirm the structural and superficial differences between these moths, naming the Columbia Basin moth *Plagiomimicusyakama* sp. n., and the southern populations with the same barcode and structure as a subspecies of it, *Plagiomimicusyakamamojave* ssp. n. When these new taxa are removed from *P.tepperi* it becomes a more uniform entity in appearance, structure, and barcodes.

No early stages of the *P.tepperi* species-group were known at the time of Poole’s revision. DLW has discovered that the larvae of these moths feed on flowers and seed heads of brickellbushes, *Brickellia* Ell. (Asteraceae). Each species appears to be a specialist on one or a few species in the genus.

TM found at SDMC another new *Plagiomimicus* species from Baja California Sur, Mexico, belonging to the same species group. It is also named herein.

The key to the Stiriini in the MONA fascicle (as Stiriinae) (Poole 1995: 81–85) is not readily modified to include the new taxa. A key to the named North American species in the *Plagiomimicustepperi* species-group is presented below. *Plagiomimicustepperi* and *P.mimica* species accounts are given in Poole (1995).

###### Key to adults of the *Plagiomimicustepperi* species-group

**Table:** 

1	Forewing reniform stigma dark and indistinct; Baja Peninsula, Mexico	*** P. incomitatus ***
–	Forewing reniform stigma a white bar or absent; United States	**2**
2	Forewing subapical spot darker than cell in medial area; right ampulla of male clasper elongate, curved, base angled strongly distad	**3**
–	Forewing subapical spot inconspicuous, lighter than cell in medial area; right ampulla of clasper of male valve short, peglike, base oriented dorsad	**4**
3	Distal postmedial area abutting subterminal line significantly darker than medial part; ampulla claspers bilaterally symmetrical, not extending distal to valve edge on either side	*** P. tepperi ***
–	Distal postmedial area abutting subterminal line not darker than medial part; ampulla of claspers asymmetrical: right longer, straight, extending beyond valve distal edge; left shorter, curved cephalad without extending distal to valve	*** P. mimica ***
4	Forewing olive and gray; Columbia Plateau of Washington and Oregon	*** P. y. yakama ***
–	Forewing ochre or ochre and tan; southern Great Basin, western Arizona, and southeastern California	*** P. y. mojave ***

###### Plagiomimicus yakama

Taxon classificationAnimaliaLepidopteraNoctuidae

Crabo & Wikle sp. n.

http://zoobank.org/D50EEB56-B99D-4FAB-8DB3-0960A8117802

Figs 11–13 , 17 , 18 , 21 , 24 , 49

####### Type locality.

USA, Washington, Yakima County, Satus Creek.

####### Type Material.

**Holotype, male.** [USA], Washington, Yakima County, Satus Creek, 30 V [19]49, E. C. Johnston. CNC. **Paratypes**. 10 males, 3 females. **USA**: **Washington**: Kittitas County: Schnebly Coulee, 46.95°N, 120.09°W, 500 m, 2 VI 1990, L G Crabo leg (4 males, 2 females); same locality & collector, 3 VII 1990 (1 male); Schnebly Coulee, 46.955°, -120.095°, 500 m, 14 V 2010, L. G. Crabo leg.; Grant County: Wanapum Dam, 1.6 mi [2.6 km] N, 46.900°, -119.948°, 250 m, 14 V 2010, L. G. Crabo leg (1 m); Yakima County: Satus Creek, 30 V [19]49, E. C. Johnston, (2 m); same locality, date, & collector / Genitalia CNC slide # 17068 (1 m); same locality, date, & collector / Genitalia slide by P6 USNM 45.585 (1 f); same locality, date, & collector / Slide No. 10,789 (1 m). CNC, DLW, LGC.

The type series is restricted to Washington State.

####### Differential diagnosis.

**Figures 11–16.**
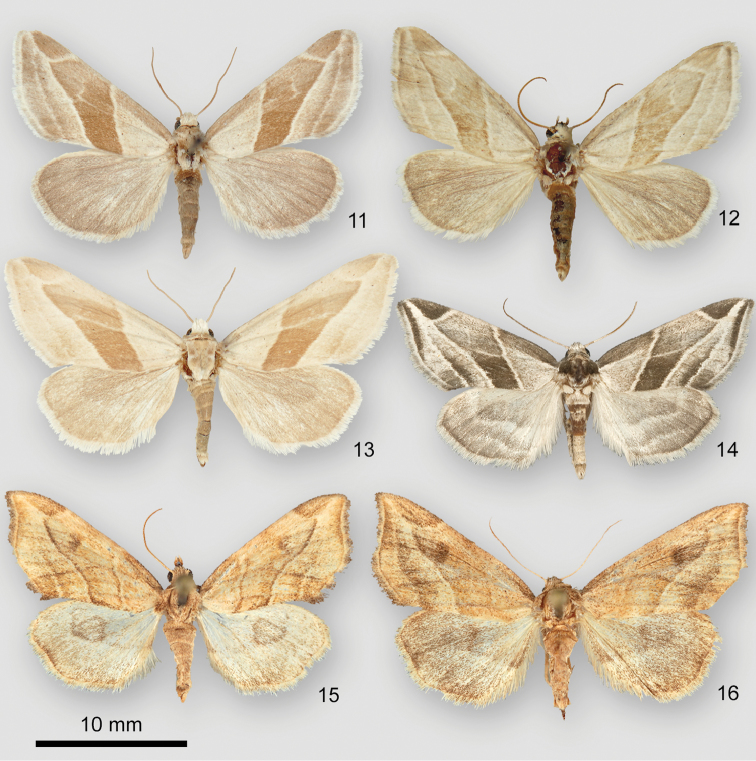
*Plagiomimicus* adults. **11***P.yakamayakama*, holotype male, USA, Washington, Yakima County, Satus Creek **12***P.yakamamojave*, holotype male, USA, Arizona, Mohave County, Hualapai Mountains, Wikieup 9.7 km W **13***P.yakamamojave*, male, USA, Nevada, Clark County, Charleston Mountains, Kyle Canyon **14***P.tepperi*, male, USA Arizona, Maricopa County, Cave Creek **15***P.incomitatus*, holotype male, Mexico, Baja California Sur, Punta Colorada 11.3 km S **16***P.incomitatus*, female, same locality as last specimen.

*Plagiomimicusyakama* (Figs 11–13) is distinguished from the two other species in the *P.tepperi* species-group found in the United States in having an inconspicuous subapical spot, not darker than the adjacent medial area. Fresh *P.tepperi* (Figure 14) and *P.mimica* (not shown) are greener than *P.yakama*. *Plagiomimicusincomitatus* sp. n. (Figs 15, 16), only found in Mexico, has a dark smudged reniform stigma unlike the pale or absent stigma of *P.yakama*.

**Figures 17–20.**
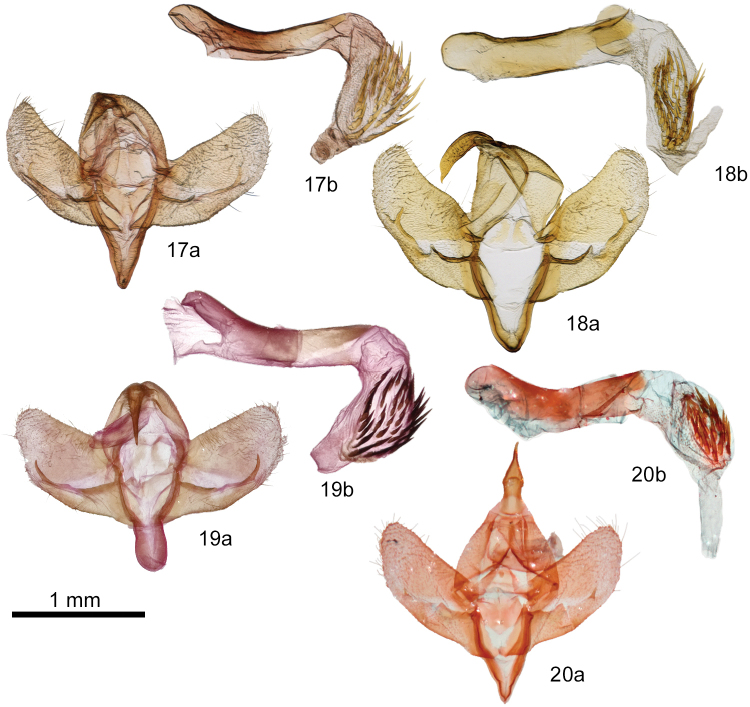
*Plagiomimicus* male genitalia. **17***P.y.yakama***a** valves **b** phallus with everted vesica **18***P.yakamamojave***a** valves **b** phallus with everted vesica **19***P.tepperi***a** valves **b** phallus with everted vesica **20***P.incomitatus***a** valves **b** phallus with everted vesica.

**Figures 21–23.**
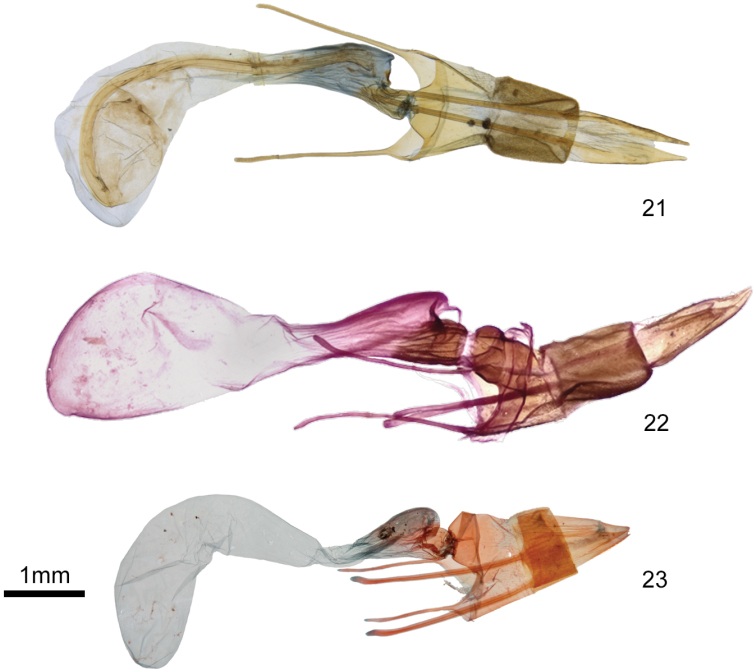
*Plagiomimicus* female genitalia **21***P.y.yakama*, female **22***P.tepperi*, female **23***P.incomitatus*, female.

The ampullae of the male valve of *P.yakama* (Figs 17a, 18a) are nearly symmetrical, short and needlelike, arising perpendicular to the valve or at a slight angle. Those of *P.tepperi* (Figure 19a) and *P.mimica* (not shown) are longer, curved, and directed distad. Those of *P.mimica* are also asymmetrical. The female of *P.yakama* (Figure 21) has a longer and more strongly curved corpus bursae than *P.tepperi* (Figure 22).

The barcodes of *P.yakama* (BOLD:ACR9301) and *P.tepperi* (BOLD:AAF2198) differ by at least 1.63 %.

####### Description.

Nominate subspecies. **Adults.** Males and females similar in size and habitus. *Head.* Antenna filiform, ventral surface ciliate, dense (male), sparse (female); dorsal scales gray olive. Scape off-white. Eye round, bare. Frontal process sideways D-shaped, straight side ventrad, lateral and dorsal rims raised slightly, central process slightly caudal to “D” center, cone shaped, protruding slightly beyond edges; a transverse ridge caudal to process; frons and dorsal head scales short, tan off-white, palest near vertex. Labial palpus reaching dorsal eye, second segment long, third segment very short, scales short, light olive gray, darker than head. Haustellum normal. *Thorax.* Dorsum, including patagium and tegula, scales short, olive off-white; appearing uniform pale brownish olive gray similar to head and forewing base. Venter lighter. *Legs*: Pale olive gray; distal foretibia claw short, thornlike, tarsal segments equal length. *Wings*: Forewing: Length 11.5–12.5 mm; elongate with slightly pointed apex, lateral margin straight to CuA1, thence convex to posterior margin; scales mixed olive off-white, tan, and gray olive; base to antemedial line and basal postmedial area silver gray, distal postmedial area, terminal area, and subapical spot slightly darker gray, medial area dark olive gray; cubital vein basal to postmedial line slightly lighter; basal and medial lines absent; antemedial and postmedial lines white, wide, slightly indistinct; antemedial line oblique from mid-costa to inner third posterior margin, slightly convex; postmedial line from outer third costa to R5 angled strongly distad, bent basad acutely on R5 to cubital vein at end of cell, thence parallel to antemedial line to outer ⅓ of posterior margin; subterminal line pale gray, white adjacent to subapical spot, slightly sinuous; terminal line thin, slightly darker than terminal area or absent; subapical spot slightly lighter than medial area, elongate, caudal margin smoothly convex; fringe olive off-white, base slightly darker; claviform and orbicular stigmata absent; reniform stigma absent or few pale anterior and darker posterior scales. Hindwing: Dorsum uniform medium gray, postmedial area slightly lighter in some specimens; terminal line thin, slightly darker; fringe whitish, base pale olive gray. *Abdomen.* Male lacking basal modifications; scales pale fuscous. *Male genitalia*: Uncus short, thick, curved slightly, point short, covered by sparse short fine setae. Juxta shield shape, height = width. Valve length 2.4–2.7 × width, simple, outer margin convex, apex blunt, medial surface with sparse fine setae; cucullus unmodified, corona absent; sacculus ⅔ × valve length, 0.4 × width, basal process short, spike like, perpendicular to valve, mesial dorsum variable, smooth or with short broad triangular process; clasper base weak, origin near ventral margin; ampulla short, 0.14–0.18 × valve width, right slightly longer, thin, acute, nearly perpendicular to valve; digitus absent. Phallus cylindrical, straight, length 4 × width; vesica as long and slightly wider than phallus, straight beyond basal 120° bend, mesial and distal surface with large patch of similar-sized basally directed spike-like cornuti. *Female genitalia*: Papilla analis 2 × segment A8 length, width 0.33 × length, sclerotized, distal ⅓ tapered evenly to acute apex, setae short, sparse, membrane to segment A8 leathery; posterior apophysis 3.3 × segment A8 length; anterior apophysis 0.8 × posterior apophysis; ostium bursae sclerotized, cup shaped, slightly wider than ductus bursae; ductus bursae 0.8 × segment A8 length, sclerotized lightly; corpus bursae elongate, length 3.5 × width, posterior ⅓ sclerotized; anterior ⅔ membranous, lacking signa, constricted mesially to width of ductus bursae, anterior end bulbous, curved slightly rightward; appendix bursae short, truncate, moderately sclerotized, joined broadly to corpus bursae at junction with ductus bursae; ductus seminalis at ventral apex.

####### Geographic variation.

Populations of *P.yakama* are arranged in northern and southern subspecies, described below.

Barcodes samples of *P.yakama* exist for Washington (*n* = 2), Utah (*n* = 3), Nevada (*n* = 1), Arizona (*n* = 6), and California (*n* = 3). Washington samples differ from the others by 0.3 %.

####### Etymology.

The name refers to the Yakama people, the original human inhabitants of the type locality of this moth. It is a noun in apposition. The spelling of the Yakama Nation differs from the more familiar spellings of the city of Yakima and Yakima County, Washington.

####### Distribution and ecology.

*Plagiomimicusyakama* is found in two separate parts of the American West (Figure 49). The nominate subspecies occurs on the Columbia Plateau. Subspecies *Plagiomimicusyakamamojave* is found 1000 km farther south in the southern Great Basin and Mojave Desert.

This moth flies in dry desert habitats with exposed soil and rocks, favoring those with varied topography such as rim rock, coulees, and arroyos. Where known, the larva of *P.yakama* feeds on *Brickellia* species. The larva of subspecies *P.y.mojave* is described below.

Adults fly during spring, from April to June, in all parts of the range. Southern populations have at least a partial fall brood.

####### Discussion.

Syntypes of *Schiniatepperi* Morrison, 1875 at MSU and the holotype of its synonym *Plagiomimicusrichii* Grote, 1886 at NHML, both described from “Texas”, match examined material of *Plagiomimicustepperi* from Texas, New Mexico, and southeastern and central Arizona. When restricted to these populations *P.tepperi* is uniform in habitus and structure. Barcodes of *P.tepperi* from southeast Arizona (*n* = 6) and New Mexico (*n* = 1) differ by 0.3 %. Barcodes of topotypical Texas populations have not been sampled. The range of *P.tepperi* extends northwards to Colorado.

In naming *P.yakama* and restricting *P.tepperi* in the above sense, the species in the *P.tepperi* species-group become more uniform well defined entities. However, ongoing rearing and molecular work by DLW and David Wagner suggest that there could be additional unrecognized cryptic species.

###### Plagiomimicus yakama yakama

Taxon classificationAnimaliaLepidopteraNoctuidae

Crabo & Wikle ssp. n.

http://zoobank.org/9C9015EB-8E77-410A-A07E-1A92FBBE4DCD

Figs 11 , 17 , 21 , 49

####### Differential diagnosis.

Subspecies *P.y.yakama* (Figure 11) is darker and greener than *P.y.mojave* (Figs 12, 13), and the pale areas of the forewing are grayer. The postmedial line of the nominate subspecies is angled slightly basad on the cubital vein, straight or slightly convex in *P.y.mojave*. Other differences are described under *P.y.mojave*. No significant differences exist in the male or female genitalia. Barcodes of the two subspecies differ by 0.3 percent, similar to intraspecies variation in *P.tepperi* and less than interspecies differences in the species-group (1.5 to 2.6 %).

####### Distribution and ecology.

The nominate subspecies occurs in the Columbia Plateau ecoregion (Figure 49) and is the most northerly of all *P.tepperi* species-group taxa. All Pacific Northwest records are from close to the 120^th^ parallel, from Vantage, Washington to southern Wheeler County, Oregon.

*Plagiomimicusy.yakama* is single brooded and flies during late spring and early summer. Its early stages are unknown, but the most likely food plant in Washington is *Brickelliaoblongifolia* Nutt. based on the presence of this plant near populations of the moth in Grant and Kittitas counties (pnwherbaria.org [accessed 23 January, 2018]).

###### Plagiomimicus yakama mojave

Taxon classificationAnimaliaLepidopteraNoctuidae

Wikle & Crabo ssp. n.

http://zoobank.org/302596E9-A32E-4AAD-B038-6AD94F479AEE

**Figure 24.**
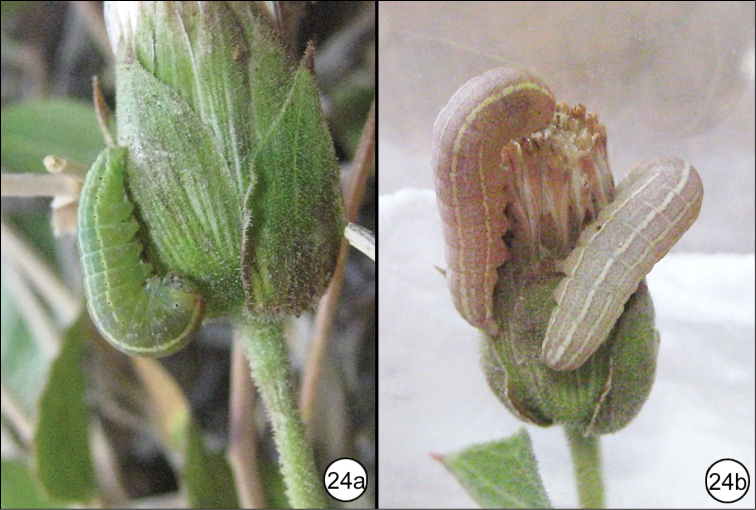
*Plagiomimicusyakamamojave*, late instars feeding on flowerheads of *Brickelliaatractyloides* (USA, Arizona, Mohave County, Hualapai Mountains) **a** penultimate instar **b** last instar.

Figs 12 , 13 , 18 , 24 , 49

####### Type locality.

USA, Arizona, Mohave County, Hualapai Mountains, 9.7 km west of Wikieup.

####### Type material.

**Holotype, male.** USA, Arizona, Mohave County, 34°40.478'N, 113°41.934'W, Hualapai Mts., 6 mi [9.7 km] W of Wikieup, 10 IX 2013, leg. D. L. Wikle, to MV / DLWC 011176 / Specimen ID CNCLEP 00116215 / Barcodes of Life Project, Leg removed, DNA extracted. CNC. **Paratypes.** 37 males, 6 females. **USA**: **Arizona**: Mohave County: Hualapai Mts, 6 mi [9.7 km] W Wikieup, 34°40.212'N, -113°42.299'W, elev 3590' [1094 m], 19 IV 2012, to B[lack] L[ight], D. L. Wikle leg. / DLWC011081 / [Crabo genitalia slide] 615 male / DNA CNCLEP 00116338 (1 m); same locality and collector as holotype, 9 V 2010 / DLWC011153 (1 m), DLWC011273 (1 m); same locality and collector, 5 IX 2012 / DLWC011018 (1 m), DLWC011039 / DNA CNCLEP 00116337 (1 m), DLWC011034 (1 m), DLWC011180 / DNA CNCLEP 00116336 (1 male), DLWC011187 (1 f), DLWC011262 (1 m), DLWC011298 (1 m), DLWC011342 (1 m), DLWC011350 (1 m), DLWC011377 (1 f); same locality and collector, 10 IX 2013 / DLWC 011126 (1 m), DLWC011291 / Specimen ID CNCLEP 00116216 / Barcodes of Life Project, Leg removed, DNA extracted (1 f), DLWC011370 (1 m); Hualapai Mts, Wikieup 9 km WSW, 34.674°, -113.699°, 1060 m, 14 IV 2015, L. G. Crabo leg. / DNA CNCLEP 00116339 (1 m); **Nevada**: Clark County: Charleston Mts, Kyle Canyon, 26 IV 1950; E. C. Johnston (12 m), Genitalia Slide, By PG, USNM 45696 (1 m), Genitalia Slide, By PG, USNM 45697 (1 m); Spring Mts, Lucky Strike Canyon Rd., 36°18.061'N, -115°29.441'W, elev. 4885' [1489 m], RRCNCA #NV-052, to BL/MV, 21 IV 2013, leg. D. L. Wikle / DLWC011299 (1 f); same locality and collector, 12 V 2013 / DLWC011294 (1 f); **Utah**: Garfield County: Old Sheffield Rd off Hwy 12, 37°43.376'N, -111°26.266'W, elev. 6005' [1830 m], 5 V 2009, leg. D. L. Wikle / DLWC011035 / Specimen ID CNCLEP 00116214 / Barcodes of Life Project, Leg removed, DNA extracted (1 m), DLWC011099 (1 m), DLWC011118 (1 m), DLWC011123 (1 m), DLWC011243 (1 m), DLWC011246 (1 m), DLWC011264 (1 m), DLWC011347 (1 f); Spencer Flat Rd mi 1.7 [2.7 km] sta 1, 37°43.411'N, -111°26.273'W, elev. 6015' [1833 m], lt B[lack] L[ight], 11 V 2012, D. L. Wikle leg., GSENM Permit#UT-12-033-01-B / DLWC011201 / [Crabo genitalia slide] 616 male / DNA CNCLEP00116333 (1 m); Wolverine Loop “draw,” 37°49.924'N, -111°6.535'W, elev. 6440' [1963 m], GSENM #UT-12-033-01-B, leg. Opler + Wikle (1 m). CNC, DLW, LGC.

The type series is restricted to Arizona, Nevada, and Utah.

####### Differential diagnosis.

Subspecies *P.y.mojave* (Figs 12, 13) is pale olivaceous ochre tan, appearing paler, yellower, and more “washed-out” than the nominate subspecies. Dark areas of the forewing are tan rather than olive, less contrasting than in *P.y.yakama* (Figure 11), and the subapical spot is vaguely darker than the adjacent wing if visible at all. Most specimens lack angulation of the postmedial line on the cubital vein resulting in a wider medial area than in *P.y.yakama*. The antemedial and postmedial lines of darker specimens are two toned, tan on the medial-area side, whereas those of *P.y.yakama* are pale. The forewing apex tends to be most pointed in this subspecies. No significant genitalia structural differences are evident between the subspecies.

*Plagiomimicusy.mojave* is most likely to be confused with *P.tepperi* (Figure 14), the western range of which approaches to within 135 km of *P.y.mojave* in northwestern Arizona. In addition to structural characters noted in the *P.yakama* description *Plagiomimicustepperi* is distinguished by slightly falcate forewing apex and a dark shade preceding the subterminal line. Differences between *P.y.mojave* and *P.incomitatus* (Figs 15, 16) are described under the latter species, though it is unlikely that these moths occur together.

####### Description.

**Adult.** Males and females similar in size and habitus. *Head.* Structure and vestiture similar to *P.y.yakama*, paler. *Thorax.* Dorsum pale yellow tan. *Wings*: Forewing: Length 11.0–14.5 mm; apex more pointed than in *P.y.yakama*; scales pale yellow and light tan; uniform pale yellowish tan outside medial area, subterminal area and subapical spot slightly darker in some specimens; medial area darker olivaceous yellow-tan, usually slightly darker on posterior ½; cubital vein slightly lighter basal to postmedial line in some specimens; basal and medial lines absent; antemedial and postmedial lines off-white, bordered by tan in medial area in dark specimens; antemedial line oblique from mid-costa to inner ⅓ of posterior margin, straight; postmedial line similar to nominate subspecies, but slightly convex near cell; subterminal line pale off-white, only visible in dark specimens; terminal line thin, tan, evident on anterior ½; subapical spot indistinct or absent, lighter than medial area when present; fringe same as terminal area; stigmata typically absent, reniform stigma occasionally a bar of few pale scales. Hindwing: Uniform pale yellowish gray, slightly darker on basal ½; fringe off-white. *Abdomen.* Paler than for *P.y.yakama*. *Male genitalia and female genitalia*: as for *P.y.yakama*.

####### Etymology.

The name refers to the distribution of this moth in and near the Mojave Desert. It is a noun in apposition.

####### Distribution and ecology.

This subspecies occurs in the Mojave Desert and southern Great Basin (Figure 49), 1000 km south of nominate *P.yakama*. Specimens have been examined from Garfield and Kane counties of southern Utah, Clark County of southern Nevada, Mohave County in northwestern Arizona, and east of the Coachella Valley in Riverside County, California. The identity of a few populations of superficially similar moths from west of the Coachella Valley remain uncertain. California specimens are therefore excluded from the type series. The apparent large separation of the ranges of the two *P.yakama* subspecies could potentially be an artifact related to limited collection in the Great Basin during the spring flight period of this species.

Larvae of *P.y.mojave* (Fig. 24a, b) have been collected and reared to adults by DLW on *Brickelliaatractyloides* A. Gray in the Hualapai Mountains of Arizona and *Brickelliaoblongifolia* in Nevada. The following description is modified from a work on the larvae of western North American moths (DL Wagner, unpublished):

Ova are placed deep inside the discoid flower heads and the early instars are internal feeders on flowers as they are going to seed. Molting occurs inside the flower head and, as the larvae progress, frass is present externally on some flowers. Larvae leave the flowers as they become spoiled, and later instars rest on stems and seed heads and feed externally on multiple flowers. The early instars are pale with red spotting and a pale supraspiracular stripe.

Penultimate instars (Figure 24a) are similar, but the ground color is green. Red spotting is reduced, remaining most prominent on the thoracic segments. The supraspiracular stripe is better developed.

The last instar is greenish red with well-defined pale mid-dorsal, subdorsal, and lateral stripes. Minute red spots develop diffusely over the course of this stage such that the larva changes from green similar to the penultimate larva initially, to more or less pink through the second half of the instar (Figure 24b). The subdorsal stripe is twice as thick as the mid-dorsal stripe. A faint diffuse supraspiracular stripe extends A1–A8. The lateral (spiracular) stripe begins at A1 and continues to the base of the A10 proleg. The spiracles are black. The primary setae are not borne from white pinacula as in other members of the *P.tepperi* species-group.

The larvae of *P.y.mojave* are similar to those of *P.mimica*, which feeds exclusively on *Brickelliacalifornica* (Torr. & A. Gray) A. Gray throughout its range. Its larva differs from *P.y.mojave* in that the lateral stripe is often cream or yellow rather than white, the subdorsal stripe is well developed, the mid-dorsal stripe is thin, and the larva is thickened through the thoracic segments.

*Plagiomimicusy.mojave* is at least partially double brooded, flying in late spring and again in early fall in areas where food plants flower in the spring and fall.

###### Plagiomimicus incomitatus

Taxon classificationAnimaliaLepidopteraNoctuidae

Mustelin sp. n.

http://zoobank.org/BC2E4B22-F5C0-4359-B28F-4FFF1F336ED4

Figs 15 , 16 , 20 , 23 , 49

####### Type locality.

Mexico, Baja California Sur, 11.3 km south of Punta Colorada.

####### Type material.

**Holotype, male.** Mexico, Baja Ca Norte [*sic*] [Baja California Sur], 7 miles [11.3 km] south of Punta Colorada, (arroyo), 23–30 XII, 1987, N. Bloomfield / USNM [genitalia slide] 46113. SDMC. **Paratypes.** 6 m, 3 f. **Mexico: Baja California Sur**: same locality, date, & collector as holotype (5 m 1 f); Same data, [genitalia slide] TM#465 (1 m); same data, [genitalia slide] USNM 46114 (1 f); Punta Colorada, 22 XII 1987, N. Bloomfield (1 f). CNC, SDMC, TM, USNM.

####### Differential diagnosis.

*Plagiomimicusincomitatus* (Figs 15, 16) can be recognized by the combination of small size (FW length 11–13 mm), pale yellow tan color, undulating brown forewing transverse lines, and S-shaped forewing outer margin with pointed apex. It is the only species in the *P.tepperi* species-group that has a large indistinct dark reniform stigma. Males (Figure 20) have short peg-like ampullae similar to those of *P.yakama*, but these are thicker in *P.incomitatus*. Females of *P.incomitatus* (Figure 23) are distinguished by having a long corpus bursae with a strong bend, but are best identified by forewing shape and maculation.

*Plagiomimicusincomitatus* has not been barcoded.

####### Description.

**Adult.** Males and females similar in habitus. *Head.* Antenna simple, venter finely setose, dorsal scales ochre. Scape pale ochre, ventral tuft short. Eye normal. Labial palpus reaching top of eye; first 2 segments long, short distal segment angled 45° rostrad; scales ochre. Haustellum normal. Frons scales tan; frontal process similar to *P.yakama*, central cone slightly longer. Dorsal head scales ochre. *Thorax.* Dorsal scales flat, bases moderately narrow, edges finely toothed, ochre, many with tan tips; patagium scales similar, slightly darker; tegula scales similar, many tan gray distally; entire dorsal thorax appearing similar to dorsal forewing; venter scales hair-like, ochre off-white. *Legs*: Foretibia apical spine short, thin; tarsal segments except apical segment with three regular rows of spiniform setae; scales tan. *Wings*: Forewing: length 11.0–12.5 mm (male), 12.0–13.0 mm (female); length 1.65 × maximum width. Apex acute, outer margin S-shaped, concave opposite cell, convex M3–CuA2. Dorsum: Scales mixed light to dark ochre and gray tan; appearing golden tan with dark dusting on costa, along veins (particularly Cu), and posterolateral medial, subterminal, and terminal areas (darkest in females), a gray-brown band in distal terminal area at concave margin; subapical spot tan, faint; basal and medial lines absent; antemedial and postmedial lines brown and cream, dark component bordering medial area; antemedial line smoothly undulating, convex at cell and fold, concave on mid-wing, angled slightly basad from costa to posterior margin; postmedial line adjacent to subapical spot pale, strongly angled distad, bent acutely basad at posterior subapical spot, thence smoothly S-shaped to posterior margin, convex from subapical spot to Cu, concave thence to posterior margin; subterminal line cream, evident mostly due to dark adjacent scales, evident adjacent to subapical patch and from M2 to posterior margin, latter segment S-shaped, parallel to postmedial line; terminal line, orbicular and claviform stigmata absent; fringe medium grayish tan, base ochre; reniform stigma brown, oval, diffuse. Hindwing: Lateral margin opposite cell weakly concave; ground color paler than forewing, whitish, dusted heavily with grayish tan scales (especially in females), darkest medial to postmedial line and near margin; discal spot diffuse, pale gray, elongate, C-shaped; postmedial line diffuse, light brown gray, anterior ⅓ perpendicular to costal margin, posterior segment perpendicular to medial margin, touching posterior discal spot; terminal line brown gray, darkest at concave margin; fringe gray off-white. *Abdomen.* scales flat, dorsum ochre tan, venter lighter. *Male genitalia*: Uncus arced, base thick, width 0.33 × length, tapered from mid-section to thin acute point. Juxta broad shield shape, height 0.67 × width. Valve flaplike, ovate, length 2.5 × width, cucullus unmodified, pointed bluntly, corona absent; sacculus 0.67 × valve length and 0.4 × width, basal process, short, triangular, median sacculus with dorsal obtuse triangular projection, shorter (1 specimen) or similar length (1 specimen) to basal process; ampulla of clasper short, 0.11 × valve width, right slightly longer and stouter than left (1 specimen) or much stouter than left (1 specimen), origin near ventral valve at end of sacculus, oriented 45° dorsad and distad to valve. Phallus tubular, length 4.5 × width; vesica length 0.7 × phallus length, width 1.5 × phallus width, bent 90° at base, bearing single median patch of numerous stout spine-like cornuti directed basad, basal cornuti absent. *Female genitalia*: Papilla analis conical, apex acute. Segment A8 length 1.2 × width. Posterior apophysis length 1.5 × segment A8; posterior apophysis 0.5 × anterior apophysis. Ductus bursae short, length 0.4 × segment A8, expanded slightly anterior to slight constriction at posterior origin; ostium bursae broad, funnel shaped. Corpus bursae elongate, length 2.8 × segment A8, slender, width 0.2 × length, anterior segment widest distal to 60° bend; appendix bursae extended posterior from broad junction to corpus bursae at junction with ductus, moderately sclerotized; ductus seminalis at ventral apex.

####### Etymology.

*Incomitatus* is Latin, meaning “unaccompanied” or “alone.” It refers both to the single known locality for this species as well as the solitude of its collector, Norris Bloomfield, during long collecting trips on behalf of SDMC in Baja California in the late 1980s.

####### Distribution and ecology.

*Plagiomimicusincomitatus* is only known from the type locality near the southern tip of the Baja Peninsula, Mexico (Figure 49). The habitat is a dry arroyo in Baja desert. All specimens were collected with black light in late December. The early stages are unknown.

####### Discussion.

*Plagiomimicusincomitatus* is classified in the *P.tepperi* species-group based the lack of the corona and basal patch of cornuti in the male genitalia. It resembles the other species in the group.

The moth fauna of the Baja Peninsula is poorly known, especially compared with that of adjacent southern California. Of what is known, much is due to Norris Bloomfield who made several collecting trips to the area during the 1980s on behalf of the San Diego Natural History Museum. Although the majority of the Noctuoidea collected by him also occur in southern California, he also encountered many species that are only known from southern parts of Arizona, New Mexico, or Texas, as well as some not known from the United States. *Plagiomimicusincomitatus* is one of the latter species, currently only known from the type locality. The apparent rarity of this moth might be due the lack of collecting in this region as well as its late December flight period.

#### Oncocnemidinae Forbes & Franclemont, 1954

##### *Sympistis* Hübner, 1823

Troubridge (2008) reviewed the genus *Sympistis* for North America north of Mexico. He named 50 new species and figured adults of 177 species. Although it is not a detailed revision of all species-groups, this paper is seminal in defining the genus. Eleven genera were placed in synonymy, including *Oncocnemis* Lederer that previously contained the majority of the species. *Sympistis* species are most numerous in western North America.

According to Troubridge (op. cit.) *Sympistis* is associated with the subfamily Oncocnemidinae by a horizontal transverse foramen across the first abdominal tergite dorsal to the tympanum. The structural features most diagnostic of the genus are found in the female: a corona of stout setae at the apex of the papilla analis and a large appendix bursae that functionally replaces the absent or small corpus bursae. The genus is diverse, and these structures are lost or modified in some species-groups. Most species have a claw-like foretibial seta, an adaptation to living in arid climates.

Male structures of *Sympistis* were not defined specifically by Troubridge other than noting that they are more uniform than those of the females. Typically, they have a simple strap-like valve with a weak sacculus. Most species-groups have a scoop-shaped distal valve lacking a differentiated cucullus but bearing a setal corona; the distal valve is bifurcated asymmetrically in the *Sympistisbadistriga* species-group. The base of the clasper is weak, but a thorn-like ampulla variably positioned along the ventral valve is a prominent feature in all species-groups. The digitus is lacking except in the *Sympistisfortis* and *Sympistischionanti* species-groups. The phallus is tubular with a bent or spiraled vesica bearing one or more elongate patches of spine-like cornuti, often with a single stout cornutus or patch of longer cornuti at the apex.

###### Sympistis ferrirena

Taxon classificationAnimaliaLepidopteraNoctuidae

Crabo sp. n.

http://zoobank.org/89FFD7D1-14EC-4897-8FFB-134A68505ED9

**Figures 25–28.**
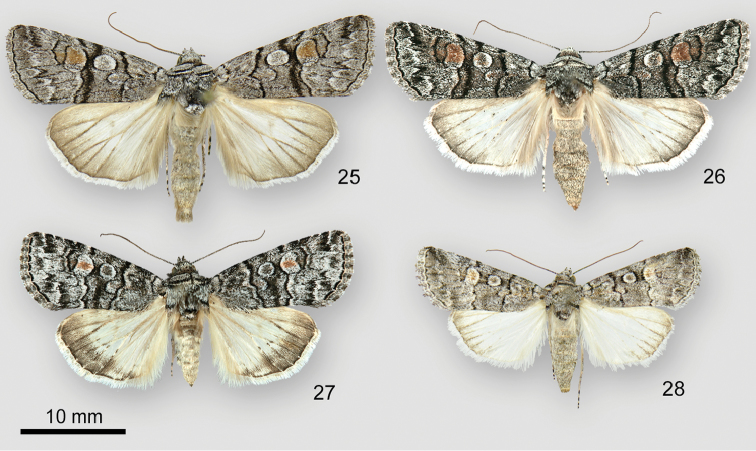
*Sympistis* adults. **25***S.ferrirena*, holotype male, USA, Arizona, Cochise County, 31°55.07'N, -109°16.54'W**26***S.ferrirena*, female, USA, New Mexico, High Rolls, Karr Canyon **27***S.dunbari*, female, USA, Washington, Whatcom County, W slope Chuckanut Mountain above Chuckanut Bay **28***S.definita*, female, USA, Oregon, Harney County, Catlow Rim.

**Figures 29–31.**
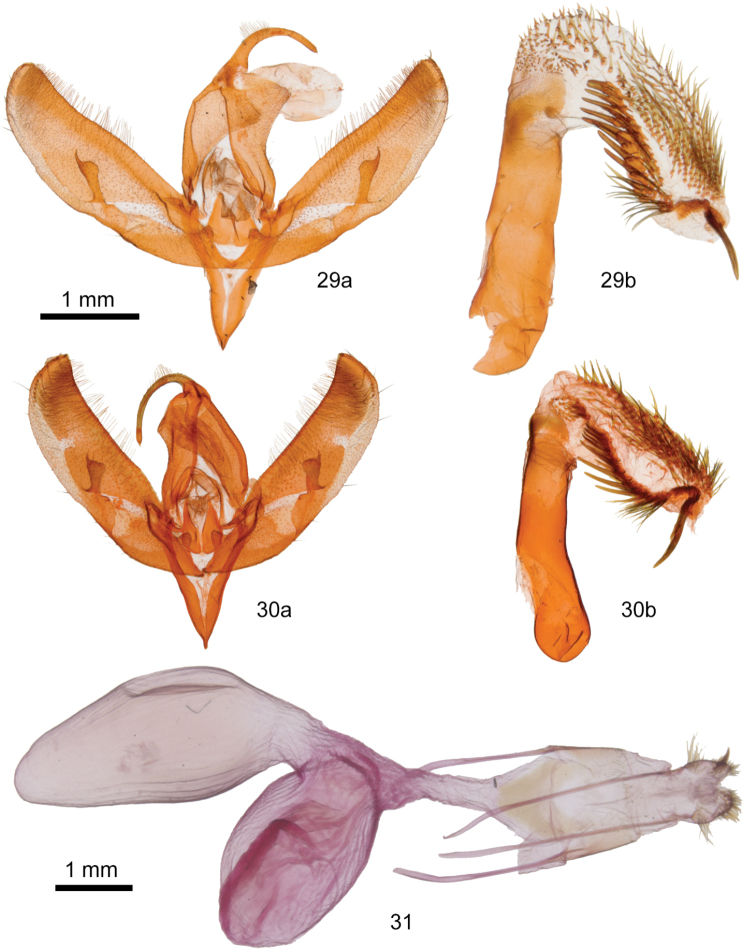
*Sympistis* genitalia. **29***S.ferrirena*, male **a** valves **b** phallus with everted vesica **30***S.definita*, male **a** valves **b** phallus with everted vesica **31***S.ferrirena*, female.

Figs 25 , 26 , 29 , 31 , 48

####### Type locality.

USA, Arizona, Cochise County, 31°55.07'N, 109°16.54'W, 2530 m.

####### Type specimens.

**Holotype, male.** [USA], Arizona, Cochise County, 31°55.07'N, 109°16.54'W, 8300' [2530 m], 18 VIII 2009, C. D. Ferris leg. / Database # CNCLEP 00080368 / Barcodes of LIFE Project Leg removed DNA extracted. CNC. **Paratypes.** 2 males, 3 females. **USA**: **Arizona**: Cochise County: Same data as holotype / Database # CNCLEP 00080369 / Barcodes of LIFE Project Leg removed DNA extracted. (1 m); Graham County: Pinaleno Mountains, Grant Creek, 8,800 ft (2682 m), 12 VII 2005, B. Walsh leg., UV light trap, Mountain stream (1 f); **New Mexico**: [Colfax County]: Sangre de Cristo Mts., Cimarron Canyon, 7900' [2408 m], 9 VII 1962, E. & I. Munroe / CNC / Genitalia slide By RS USNM 43,457 (1 m); same locality & collectors, 10 VII 1962 / Genitalia slide By RS USNM 43,458; Otero County: High Rolls, Karr Canyon, 32.898°, -105.813°, 2400 m, 9 VI 2016, L. G. Crabo leg. (1 f). CNC, LGC, TM.

####### Differential diagnosis.

*Sympistisferrirena* (Figs 25, 26) resembles *S.dunbari* (Figure 27) and *S.definita* (Figure 28). *Sympistisdefinita* occurs predominantly in the Great Basin and Colorado but has been collected with *S.ferrirena* in Arizona. *Sympistisdunbari* occurs west of *S.ferrirena* near the West Coast. *Sympistisferrirena* is slightly larger (forewing length greater than 14 mm in *S.ferrirena*, less in the other two species) and can be distinguished by its smooth thick black transverse lines and large reniform stigma with brick-red filling. The antemedial and postmedial lines of *S.ferrirena* are straight or curved smoothly from the cubital vein to the posterior margin and lack an outer black component. The corresponding lines of *S.dunbari* and *S.definita* are toothed on the veins and double with two black components. The reniform stigmata of these species are smaller and contain fewer reddish scales than that of *S.ferrirena*.

The male genitalia of *S.ferrirena* (Figure 29) and *S.definita* (Figure 30) are similar. The distal valve of *S.ferrirena* tapers more than that of *S.definita*. The sacculus of *S.ferrirena* is narrower relative to valve length: 0.10 × compared to 0.16 × for *S.definita*. The anterior (concave surface) cornuti on the vesica of *S.ferrirena* are longer and much stouter than those of the posterior patch; this distinction is less dramatic in *S.definita*.

The female corpus bursae and appendix bursae of *S.ferrirena* (Figure 31) are similar in size and shape. In *S.dunbari* and *S.definita* the appendix bursae is smaller and more curved than in *S.ferrirena*.

The DNA barcode of *S.ferrirena* (BOLD:AAU2696; *n* = 3) differs from those of *S.dunbari* (BOLD:AAE1138) and *S.definita* (BOLD:AAE5103) by at least 4.0 %.

####### Description.

**Adults.** Males and females similar in size and habitus. *Head.* Antenna filiform, venter densely pubescent, segments with single short anterior and posterior seta, dorsal scales sparse, gray. Scape untufted, scales even length, black or white. Eye normal, adjacent scales except near palpus long, lashlike, dark gray. Labial palpus scales mixed black and gray; flat laterally, long hair-like anteriorly; third segment length 0.3 × second segment, scales very short. Haustellum normal. Frons flat, scales short, mixed gray and off-white. Dorsal head scales narrow, bifurcate, mixed gray, white, and white tipped black; weak paramedian tufts anterior and dorsal to antenna. *Thorax.* Patagium scales mixed black, white, and white tipped light gray, truncate, fine toothed; dorsum appearing medium gray with black and light gray transverse stripes; tegula and dorsum scales mixed black- and white-tipped medium gray strap-like and black hair-like; appearing hoary with black-edged tegula and black weak metathoracic median tuft. Ventral thorax lighter gray, scales hairlike. *Legs*: Distal foretibia with claw-like seta over basitarsus; mid- and hindtibia lacking stout setae; scales spatulate, mixed gray, black, and white. Tarsal segments except distal segment with three rows of ventral spiniform setae, scales mixed black and gray, distal ring irregular, white. *Wings*: Forewing: Length 14.0–15.5 mm; length 2.0–2.1 × width, apex not pointed strongly, outer margin scalloped weakly, nearly straight from apex to CuA2, then curved slightly to trailing margin; dorsal scales mixed white, medium gray, dark gray, black, and dark umber, appearing hoary; basal line black, thin except tooth distad on R, curved to end at wing base on Cu; antemedial line light gray inner, thick black outer components, gull wing shape, apex basad on Cu; medial line black, thick, blurred, angled distad from mid-costa to orbicular and reniform stigmata; postmedial line components black inner, thinner than antemedial and medial lines, pale gray outer, costal origin near medial line, quadrate distad around reniform stigma, crossing posterior stigma, bent sharply caudad medial stigma to margin near medial line; postmedial line whitish gray irregular chevrons, preceded by long, thin dark, gray, intervenal wedges; terminal area diffuse black spots near costa and CuA2; terminal line black, thickest between veins; fringe scales mixed white and pale base dark gray; stigmata black lined with pale gray; claviform stigma reduced to curved black tip; orbicular stigma oval, moderately large, filling whitish gray, center slightly darker; reniform stigma ovoid, moderately large, weakly kidney shaped, thickest medially, filling diffuse, rust red. Hindwing: Outer margin straight from Rs to bend between M3 and CuA1, thence straight to anal angle; dorsum whitish gray, slightly opalescent, margin suffused medium gray; veins, discal spot, terminal line darker gray; fringe white, base striped yellow and gray. *Abdomen.* Scales flat, hair-like, medium gray; no tufts. Male with hair-like coremata from rugose patch at end of sclerotized band in membrane lateral to sternite A1. *Male genitalia*: Tegumen widest lateral to uncus base. Uncus thin, cross-section triangular, arced, tip acute. Juxta triangular, apex notched at phallus base, height 1 × width. Valve straplike, widest mesially, tapered slightly to both ends, length 3.6 × width, cucullus convex, lacking “neck,” corona of ~ 20 claw-like setae; sacculus 0.5 × length, 0.36 × width of valve, width nearly even except slight broadening at base and dorsal notch near clasper ampulla; clasper base weak, ampulla origin at mid-valve near ventral margin, directed dorsad and 45° distad, length 0.7 × valve width, straight, clubbed with rounded apex bearing cluster of small setae and thorn-like extension from lateral side; digitus absent. Phallus tubular, length 4.2 × width. Vesica 0.8 × phallus length, straight beyond basal 135° ventral bend; bearing stout apical cornutus 0.25 × phallus length, long broad band from dorsal distal phallus to posteroventral apex of innumerable cornuti, lengthening gradually from short, spike-like at base to quill-like distally, slightly smaller patch on rostrodorsal mid- to distal vesica of fewer, ~ 40, longer, stouter cornuti in parallel rows, and an apical tuft of thinner similar-length cornuti. *Female genitalia*: Papilla analis blunt tipped, trapezoidal, length 1 × basal width, covered moderately densely with fine hair-like setae, densest apically, longest basally; subapical “corona” loose double row of uneven stout, blunt spike-like setae curved slightly away from midline, longest 6 × as long as wide. Segment A8 width 1.5 × length, sparse very fine hair-like setae densest and longest at posterior and inferomedial margins. Posterior apophysis length 3.5 × segment A8; anterior apophysis length 0.7 × posterior apophysis. Ductus bursae length 2.5 × segment A8 length; ostium bursae width 0.67 × segment A8 width, ventral margin sclerotized lightly; posterior ⅓ ductus bursae triangular, tapered anteriorly, anterior segment membranous, tubular. Corpus bursae bisaccate, U-shaped, length 5 × segment A8 length, main part midline, posterior ⅓ tubular with broad attachment to appendix bursae on right, anterior ovate, length 2 × width; appendix bursae length 0.6 ×, width 1 × corpus bursae, elongate, ovate, base perpendicular to corpus bursae, distal ⅓ bent 90° ventrad, ductus seminalis slightly proximal to posterior apex.

####### Etymology.

The name is from the Latin *ferrum*, meaning iron, and *renis*, meaning kidney. It refers to the prominent rust-red filling of the reniform stigma of this moth.

####### Distribution and ecology.

*Sympistisferrirena* occurs in Arizona and New Mexico (Figure 48). It has been collected at middle to high elevations within a limited range between 2400 and 2700 meters. The habitat is mixed forest.

The flight period of adults is from June to July. Like most temperate species in the genus, it is nocturnal and comes to light. The early stages are unknown.

#### Noctuinae Latreille

##### Xylenini Guenée, 1837

###### Xylenina Guenée, 1837

####### *Aseptis* McDunnough, 1937

Mustelin and Crabo (2015) revised the genus *Aseptis* McDunnough. It contains 16 species from the United States and northern Mexico west of the Great Plains, with the greatest diversity in California. *Aseptis* species occur most commonly in steppe, desert, or shrub habitats.

Most *Aseptis* species are fairly nondescript, medium-sized (wingspan 27.5–45.0 mm), gray or gray-brown moths. Despite their dull appearance they can usually be identified as belonging to the genus by the combination of eyes devoid of setae and the presence of a concave segment of the hindwing margin between veins M1 and M3. Many *Aseptis* species, including the one described herein, have a pale postreniform patch distal to the reniform stigma that is more conspicuous than the stigma.

Structural features of the genus are detailed in the revision. Males have a thin curved uncus, a strap-like valve with weak sacculus, simple curved ampulla of the clasper, a thin pointed digitus oblique to the valve, and a weak cucullus bearing a simple corona. The vesica of the phallus is bulbous with a stout distal cornutus directed basad. Females have a triangular papilla analis, a membranous ductus bursae, and a bilobed corpus bursae with a sack-like or weakly bilobed appendix bursae.

A key to *Aseptis* adults was presented in Mustelin and Crabo (2015: 62–65). Males of *A.harpi* sp. n. will key out to Couplet 11 and females to Couplet 21. In order to include all *Aseptis* species the key can be modified by replacing those couplets with the following:

**Table:** 

11	Claviform stigma short, not extending distal to mid-medial area; subterminal line ochre to tan, undulating, not extended on veins; widespread in western North America	*** A. binotata ***
–	Claviform stigma long, nearly reaching postmedial line; subterminal line with whitish W-marks to margin below apex and on veins M3 and CuA1; Arizona, California, Utah	**11a**
11a	Forewing postmedial line posterior to reniform stigma faint, when visible filled with similar color as adjacent medial and postmedial areas; digitus of male valve oriented 50° to valve; Arizona and California	*** A. susquesa ***
–	Postmedial line prominent below reniform stigma, filling whitish, paler than adjacent areas; digitus oriented 30° to valve; Utah	*** A. harpi ***
21	Apex of papilla with a thin sclerotized flange; dorsal forewing ground color dull brown; California	*** A. perfumosa ***
–	Dorsal papilla with or without apical small tooth-like process; if present, forewing not dull brown; multiple western states, including California	**21a**
21a	Papilla analis with small apical tooth-like projection AND dorsal forewing mottled gray and orange brown; Utah	*** A. harpi ***
–	Papilla with or without tooth; if present, forewing not gray and orange brown; multiple western states including Utah	**22**

######## Aseptis harpi

Taxon classificationAnimaliaLepidopteraNoctuidae

Crabo & Mustelin sp. n.

http://zoobank.org/2F4866F8-6768-40B5-AFF3-C7426A2A6813

**Figures 32–34.**
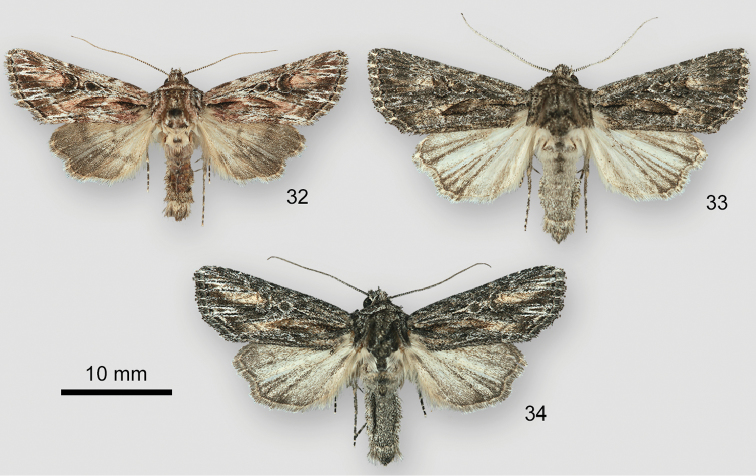
*Aseptis* adults. **32***A.harpi*, male, USA, Utah, San Juan County, 1.6 km N of Bluff, **33***A.serrula* male, USA, Arizona, Maricopa County, Cave Creek **34***A.susquesa*, male, USA, Arizona, Mohave County, Wikieup 9 km WSW.

**Figures 35–40.**
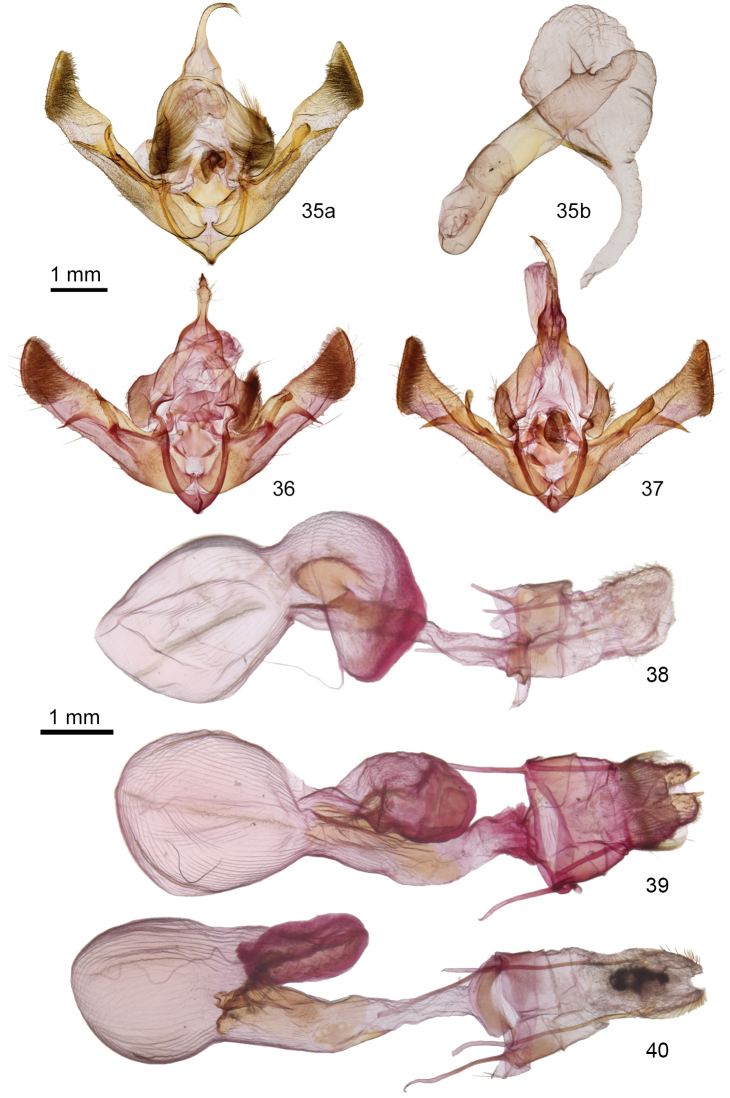
*Aseptis* male and female genitalia. **35***A.harpi***a** valves **b** phallus with everted vesica **36***A.susquesa*, male, valves **37***A.serrula*, male, valves **38***A.harpi* female **39***A.serrula* female **40***A.susquesa* female.

Figs 32 , 35 , 38 , 48

######### Type locality.

USA, Utah, San Juan County, 1.6 km north of Bluff, 1336 m.

######### Type material.

**Holotype. Male.** [USA], Utah, San Juan County, 37.2940°N -109.5656°W, 1 mi. [1.6 km] N Bluff, W. 3^rd^ St. above Cottonwood Crk., *Ericameria*/*Atriplex* hab[itat], 13 May, 2016, 4382' [1336 m.] elev., Chuck Harp / Specimen ID CNCLEP00140353 / Barcodes of Life Project, Leg removed, DNA extracted. CNC. **Paratypes.** 14 males. **USA**: **Utah**, Emery County: NW of Goblin Valley S[tate] P[ark], San Rafael Desert, 15–16 V 2007, at uv trap, pinyon-juniper desert shrub, 5300' [1615 m] elev., 38.6677°, -110.6293°, Chuck Harp leg. (1 m); Garfield County: Capitol Reef N[ational] P[ark], Pickaboo R[a]ng[e]r. St[atio]n., 2 VI 1994, M[ercury] V[apor]L[ight], P. A. Opler [leg.], / [CNC] Slide male No. 11,942 / Specimen ID CNCLEP00140316 / Barcodes of Life Project, Leg removed, DNA extracted (1 m); San Juan County: same locality, date, & collector as holotype / Specimen ID CNCLEP00140354 / Barcodes of Life Project, Leg removed, DNA extracted (1 m); same locality, date, & collector as holotype (8 m); same locality & collector as holotype, 22–23 V 2017, taken at blacklight, blackbrush/*Ericameria*/*Atriplex* (5 m, 1 f). CH, CNC, CSUC, DNHC, LGC, TM.

######### Differential diagnosis.

*Aseptisharpi* (Figure 32) is most likely to be confused with *Aseptisserrula* (Barnes and McDunnough, 1918) (Figure 33) and *Aseptissusquesa* (Smith, 1908) (Figure 34). Both of these similar species are from the deserts of southern California, southern Nevada, and Arizona. Although neither species is known currently from Utah, they could potentially occur with *A.harpi* near the Arizona-Utah border.

DNA barcodes suggest that *A.serrula* is the closest relative to *A.harpi*. Males are distinguished easily because the antenna of *A.harpi* is filiform, whereas that of *A.serrula* is serrate. *Aseptisharpi* has a lighter, more mottled, and more colorful forewing than *A.serrula*, with patches of pale gray and orange tan rather than powdery dark gray. The hindwing of *A.harpi* is gray distal to the postmedial line, whereas the entire hindwing of *A.serrula* is whitish.

Superficially, *Aseptisharpi* most closely resembles *Aseptissusquesa*. Both species have narrow male antennae and forewings with patches of orange brown. The forewing spots and postmedial and subterminal lines of *A.harpi* are more sharply defined and conspicuous than in *A.susquesa*. Pale filling of the postmedial line and whitish “W” marks of the subterminal line on veins below the apex and on M3 and CuA1 are particularly prominent in *A.harpi*. Hindwing color differences between these species are similar to those between *A.harpi* and *A.serrula*.

The male genitalia of *A.harpi* (Figure 35), *A.serrula* (Figure 36), and *A.susquesa* (Figure 37) are similar. The angle between the digitus and the valve is narrower in *A.harpi* than in the others, approximately 30° in *A.harpi* compared to nearly 50° in the other species. The digitus of *A.harpi* extends a shorter distance below the valve than in the other species.

Females of can be identified by characters of the papillae anales. The papilla analis of *Aseptisharpi* (Figure 38) has a single short apical process and lacks long basal setae. That of *Aseptisserrula* (Figure 39) has a finger-like apical process with adjacent scale-like tubercles and a dense basal collar of long seta. *Aseptissusquesa* (Figure 40) lacks an apical process and has few very long setae at the base. The appendix bursae of *A.harpi* is longer than those of the other two species.

The barcode of *A.harpi* (BOLD:ADH0685; *n* = 2) differs from those of *A.serrula* and *A.susquesa* by approximately 5 %. *Aseptisharpi* and *A.serrula* form a sister pair closest to *Aseptiscatalina* (Smith) on a similarity tree. Major haplotypes of other *Aseptis* species are listed in Mustelin and Crabo (2015: 59–60).

######### Description.

**Adult.** Males and females similar in habitus. *Head.* Antenna filiform, ventral male antenna densely setose; dorsal segments barred, scales gray, pale yellowish tan. Scape off-white. Eye normal. Haustellum normal. Labial palpus first and second segments similar, third segment short, porrect; scales short, off-white and gray. Frons smooth; scales strap-like, off-white on lower ⅔, mixed off-white and gray on dorsal ⅓, forming slight median ridge. Dorsal head scales longer, strap-like, mixed off-white and gray, sculpted weakly anteriorly and on vertex. *Thorax.* Dorsal scales longer and broader than on head, weakly spatulate, edges finely serrate, mixed tan, off-white, gray, glossy black; appearing powdery brownish tan with irregular dark and light bands on patagium and tegula, metathorax with weak dark tufts. Venter scales white and gray. *Legs*: Tibiae lacking spines, scales mixed gray, off-white, darker than venter; tarsi except apical segment with three irregular rows of spine-like setae, gray, off-white distally. *Wings*: Forewing: length 12.5–13.0 mm (males); 14.5 mm (female), length 2.25 × width, apex more pointed than in other *Aseptis*species, outer margin angled slightly on vein M3; dorsal scales three- and four-toothed, white, light yellow, tan, orange tan, light brown, dark brown, or black; appearing mottled gray brown with light orange brown postreniform patch and in fold near claviform stigma; veins thin, black, terminal R5, M1, M3, CuA1, A1+2 lined on each side with whitish scales; basal line a long dark spot on costa; antemedial line a dark spot on costa, pale posterior to cell, strongly convex on 1A+2A; medial line brown, indistinct, costa to reniform stigma; postmedial double, dark and light spots on costa, faint dark lines across postreniform patch, double dark lines with whitish filling from M3 to posterior margin, angled 45° distad from costa, bent 90° basad on M2 to posterior margin; subterminal line absent; terminal line black, absent at apex; fringe dark gray, base yellow, light checkering at veins; basal dash black, acute, to antemedial line; claviform stigma black, base broad, tip to distal medial area, acute, filling same as adjacent ground; orbicular stigma elongate, oval, thin, black, double, pale gray between lines, black centrally; reniform stigma medium sized, asymmetrically kidney-shaped, largest posteriorly, thin, black, double, filling between lines light gray basally, orange brown distally, center dull black. Hindwing: margin undulating, concave strongly M1–M3 and weakly CuA2–2A; dorsum light gray tan, darker fuscous distal to postmedial line except at inner margin; discal spot and postmedial line slightly darker gray, postmedial line indistinct, undulating; fringe orange brown, edge whitish. *Abdomen.* Male base with brush-like coremata and pockets; scales fuscous; weak median dorsal tufts on segments A1–A3. *Male genitalia*: Uncus slightly flattened at base, cylindrical distally, arced, acute tip hooked slightly downward. Tegumen with broad penicillus lobes. Juxta base broad, narrowing toward base of phallus, height 0.8 × width. Valve length 5.25 × width, strap-like, mid-portion slightly wider due to costa bulge; sacculus small, weak, 0.25 × valve length, 0.8 × width; clasper base weak, ampulla 1 × valve width, rod-like, apex blunt, origin at mesial mid-valve, base directed dorsad and 45° distad, arced with distal portion parallel to dorsal valve; digitus base near ampulla, directed distad and 30° caudal, length 0.9 × valve width, straight, narrow, acute, apex just caudal to valve margin; cucullus 1.75 × valve width, asymmetric, apex elongate, pointed bluntly, anal angle rounded, “neck” weak, corona single row of ~ 30 claw-like setae, longest near apex. Phallus cylindrical, length 4.5 × width. Vesica ~ 1.5 × phallus length, expanding gradually to 2 × width beyond mid-point, curved 180° to end ventral and slightly left of mid-phallus; sub-apex with broad dome-shaped diverticulum and prostrate, rodlike, basally-directed cornutus 0.5 × phallus length opposite diverticulum. *Female genitalia*: Papilla analis truncate, longest dorsally, very small tooth-like process at medial dorsal tip, sparse short hair-like setae densest on dorsum and apex, longest basally without dense “corona.” Posterior apophysis length 2.5 × segment A8; anterior apophysis 0.8 × posterior apophysis. Segment A8 length 0.67 × width, glabrous. Ostium bursae membranous, ventral lip sclerotized, band-like; ductus bursae length 4.5 × segment A8, tubular, posterior ⅔ membranous, anterior ⅓ sclerotized except membranous anterior ventral and right sides; corpus bursae 5 × segment A8 length, ovoid, width 0.6 × length, long signa evenly spaced on anterior, posterior, and lateral sides; appendix bursae length ~ 1 × corpus bursae length, narrower, projecting slightly leftward from origin dorsal to junction with ductus bursae, curved to end ventral and to left of ductus-corpus junction; ductus seminalis at anterior apex.

######### Etymology.

The eponym honors Chuck Harp of Littleton, Colorado who recognized this moth as an undescribed species and brought it to our attention. Most of the known specimens of this species have been collected by him.

######### Distribution and ecology.

*Aseptisharpi* has a limited range in eastern and southeastern Utah (Figure 48). It has been collected in the red rock country of Garfield, Grand, and San Juan counties. The habitat is shrub steppe. Collection dates are from May and early June.

The early stages are unknown. The larva is probably a climbing cutworm that feeds on woody shrubs based on the habits of other *Aseptis* species (Mustelin and Crabo 2015).

######### Discussion.

The discovery of this species is a surprise to us since we revised *Aseptis* recently (Mustelin and Crabo 2015). No new species were recognized at the time, although two new genera were described and the number of recognized species was reduced significantly.

##### Eriopygini Fibiger & Lafontaine, 2005

###### *Hypotrix* Guenée, 1852

*Hypotrix* Guenée is a moderately large genus of New World moths distributed from the American Southwest to South America. The thirteen previously known species in the United States were revised less than a decade ago (Lafontaine et al. 2010). The genus is diverse, both in external appearance and structure, and is difficult to define concisely. The most diagnostic character is the female papilla analis, swollen basally with rapid taper to a point. The eye is covered with hairs similar those of most genera in the Eriopygini. Tibial spines are variable between species. Males often have brushes on sternites A1 and A8. The uncus is expanded. The distal valve has either a triangular cucullus demarcated from the valve by a ventral notch and bearing a corona and ventral spine, or has a reduced cucullus with a vestigial or absent corona. The sacculus has a sclerotized ventral part and a membranous ventral flap that overlaps adjacent structures. The vesica of the phallus is long, coiled, and bears several groups of cornuti. In addition to the distinct papilla analis, the female has a sclerotized tubular ductus bursae, a membranous bulbous corpus bursae, and a long spiraled appendix bursae (op. cit.).

A fourteenth American *Hypotrix* species from Arizona is described below. This moth, although similar structurally to *Hypotrixhueco* (Barnes, 1904), is strikingly patterned olive green and pure white, unlike any other species in the genus.

####### Hypotrix lactomellis

Taxon classificationAnimaliaLepidopteraNoctuidae

Wikle & Crabo sp. n.

http://zoobank.org/DFE33E03-F671-464C-B583-EE44739C8874

**Figures 41–43.**
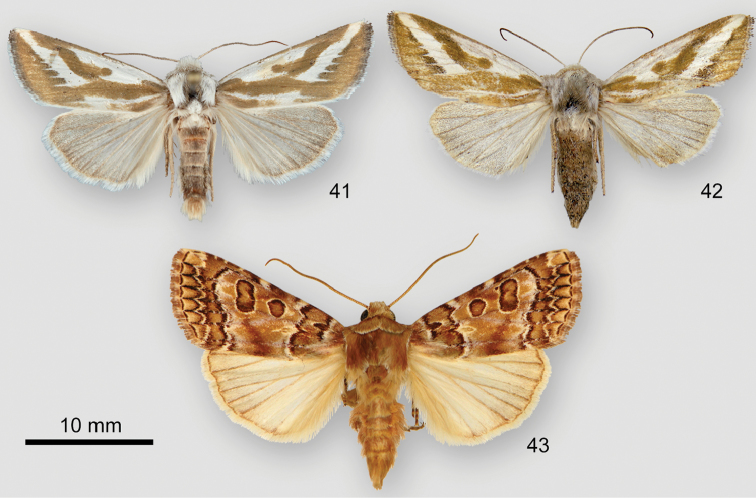
*Hypotrix* adults. **41***H.lactomellis*, male, USA, Arizona, Apache County, Eagar, 11.1 km W, South Fork Road at Little Colorado River **42***H.lactomellis*, female, USA, Arizona, Apache County, White Mountains, South Fork Road **43***H.hueco*, male, USA, Arizona, Cochise County, Huachuca Mountains, Ash Canyon.

**Figures 44–47.**
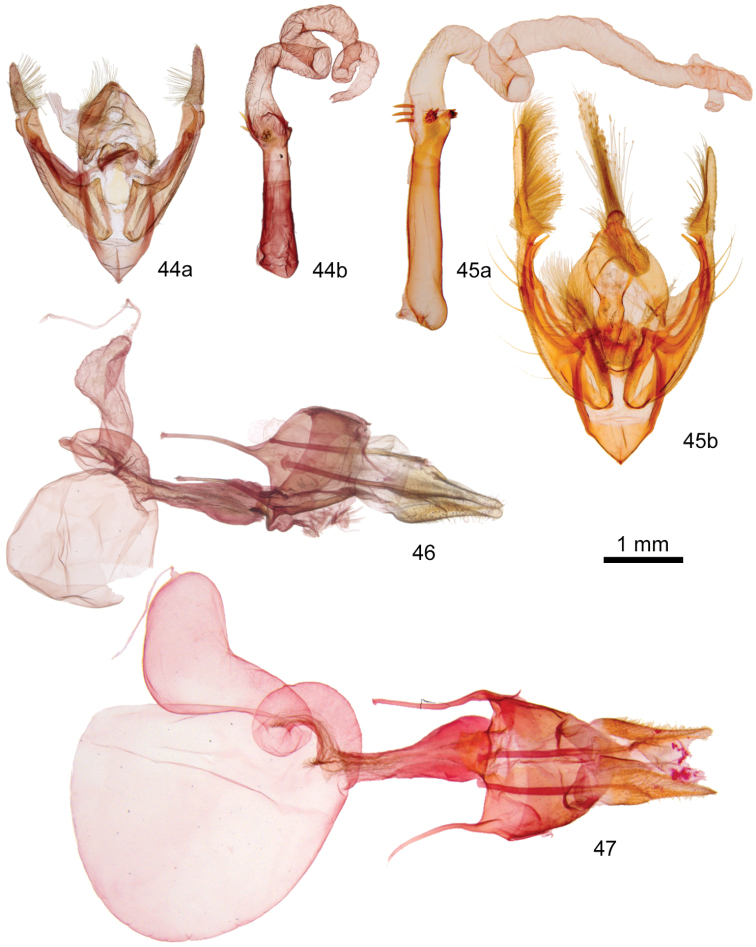
*Hypotrix* genitalia **44***H.lactomellis*, male, **a** valves **b** phallus with everted vesica **45***H.hueco*, male, **a** phallus with everted vesica **b** valves **46***H.lactomellis*, female **47***H.hueco*, female.

Figs 41 , 42 , 44 , 46 , 49

######## Type locality.

USA, Arizona, Apache County, White Mountains, Whiting Knoll, FR117, 2766 m.

######## Type specimens.

**Holotype, male.** USA, Arizona, Apache County, White Mts, Whiting Knoll, FR117, 34°9.580'N, 109°34.516'W, elev. 9075' [2766 m], 6 VII 2007, MV, leg. D.L. Wikle. CNC. **Paratypes**: 47 males, 12 females. **Mexico**: **Coah**[**uila**]: nr. Jame, 33 mi [53.1 km] S.E. Saltillo, 7500' [2286 m], 18 VII [19]63, H. and A. Howden / Database # CNC LEP 00094171 / Genitalia CNC slide 16649 male (1 m); **USA**: **Arizona**: Apache County: Eagar, 6.9 mi [11.1 km] W, South Fork Rd @ Little Colorado [River], 34°05'19.5"N, -109°24'48.76"W, 22–23 VII [20]12, Leg. M. L. Raschko. Light trap (2 m, 1 f); Greer, 20 VI 1986, R. & J, Robertson / Database # CNC LEP 00031876 / Genitalia CNC slide # 16650 male (1 m); Greer, 33°59'50.88"N 109°27'50.73"W, elev. 8450' [2576 m], 3 VII 2013, Leg E. Rand (1 f); same locality & collector, 18 VII 2013 (2 m); White Mts., South Fork Rd., 34°5.371'N, -109°24.599'W, elev 7370' [2246 m], 22 VII 2012, to B[lack]L[ight], M. Raschko leg. (12 m 4 f); same locality, date, & collector, DLWC11043 PARATYPE female / Specimen ID CNCLEP 00113790 / Barcodes of Life Project, Leg removed, DNA extracted / Genitalia CNC slide # 17432 female (1 f); same locality, date, & collector / Specimen ID CNCLEP 00140416 / Genitalia CNC slide 17433 female (1 f); same locality, date, & collector, DLWC11038 PARATYPE male / Specimen ID CNCLEP 00140415 (1 m); same locality, date, & collector, DLWC 11043 PARATYPE female / Database # CNCLEP 00113790 / Barcodes of Life Project, Leg removed, DNA extracted / Genitalia CNC slide # 17805 female (1 f); Little Colo[rado] R[iver], South Fork Rd, 34°5.371'N, 109°24.599'W, elev. 7370' [2246 m], 25 VII 2013, to BL, leg. D.L. Wikle (1m 2f); South Fork of Little Colorado River, 34°05'19.82"N, 109°24'39.43"W, elev. 7360' [2243 m], 19 VII 2013, Leg E. Rand (2 m); same locality & collector, 28 VI 2014 (4m 1f); Same data as holotype (3 m); White Mts, 3 mi [4.8 km] S Water Cyn, 34°3.976'N, 109°17.571'W, elev. 7500' [2286 m], BL, 22 VII 2012, leg. M. Raschko (1 m); Benny Creek, 34°02'27.16"N, 109°27'25.69"W, elev. 8250' [2515 m], 21 VI 2017, Leg E. Rand (20 m); **New Mexico**: Catron County: Quemado, 8 mi. [12.9 km] S., 27 VI 1987, elev. 7200' [2195 m], Acc. #1117, P.M. Jump (1 m). CNC, DLW, ERLGC, MLR.

######## Differential diagnosis.

*Hypotrixlactomellis* sp. n. (Figs 41, 42) is a distinctive moth. No other species in the tribe Eriopygini has a similar olive and pure white forewing. It is more likely be confused with a *Schinia* Hübner (Heliothinae), such as the silver and olive-gray species allied to *Schiniacumatilis* (Grote), or a species of Acontiinae. The eye of *Hypotrix* is hairy, lacking hairs in these look-alikes.

Structurally, *H.lactomellis* is most similar to *Hypotrixhueco* (Figs 43, 45, 47). Despite the close relationship suggested by the genitalia, these moths could not be more un-alike in appearance. *Hypotrixhueco* is mottled red brown like many moths from pine forest habitats.

The barcode of *H.lactomellis* (BOLD:ACO7143) is most similar to that of *H.hueco* (BOLD:AAI8440), differing by 4.5 %.

######## Description.

Adults. Males and females similar in habitus. *Head.* Male antenna filiform, ventral surface densely setose; female antenna with fewer setae; dorsal scales off-white, light gray basally. Scape white. Eye normal size, interfacetal setae short, adjacent scales light gray, few hair-like black. Labial palpus scales white, gray, darkest distally, flat on sides, hair-like anteriorly. Haustellum normal. Frons rounded, scales off-white. Dorsal head scales hair-like, white. *Thorax.* Scales mixed hair-like and thin bifurcate; patagium olive off-white; tegula white, a few scales on central portion olive-tipped; central dorsum light olive; metathoracic tuft white, weak. *Legs*: Lateral tibiae lacking spines or other modifications. Tarsal segments except apical segment with 3 irregular rows of ventral spiniform setae. *Wings*: Forewing 12.0–13.0 mm (males), 12.5–13.0 mm (females), length 2.3 × width, apex pointed, outer margin nearly straight from apex to CuA2, then curved basad; dorsal scales mixed white, olive off-white, and olive; appearing uniform greenish olive patterned with white, transverse lines and spots contributing pattern borders but not discernable otherwise; three white areas: 1) Cell except fused olive orbicular and reniform stigmata, white extending to base as line on R; 2) Oblique even-width band from apex toward mid-posterior margin, ending at fold, white extending into postmedial area as lines on M2, M3, CuA1, CuA2; 3) Medial area posterior to fold, extending to base and into subterminal area on 1A+2A; claviform stigma absent; orbicular and reniform stigmas olive green, fused; orbicular stigma small, oval; reniform stigma elongate, weakly S-shaped, oblique, tilted and extended toward apex; fringe olive. Hindwing: Dorsum light tan gray, gradually darker gray to outer margin; veins and terminal line thin, gray; discal spot absent; fringe white, base light yellow. *Abdomen.* Scales mostly light tan flat or hairlike, weak white median tuft on dorsal segment A1. Male lacking coremata, pockets, brushes. *Male genitalia*: Uncus base cylindrical, apex flattened to blunt rhomboid tip, 1.67 × basal width, with slight dorsal median crest, distal half with long hair-like setae, shortest beneath tip. Juxta elongate shield shape, height 1.5 × width. Valve length 3.4 × basal width, tapered from base to apex, elongate triangular pointed cucullus demarcated by triangular notch in ventral valve margin, medial surface with numerous hair-like setae, corona and spine absent; sacculus 0.6 × valve length, mesial surface flaplike, overlapping clasper base; clasper base long, sclerotized, lacking defined dorsal and ventral divisions, ampulla oriented distad and slightly ventral to valve axis from origin just past mid-valve, horn shaped, curved to overlap digitus base to end at cucullus base; digitus directed ventrad from origin posterior to proximal ampulla, semicircular, extending just ventral to valve. Phallus length 3.8 × width, tubular, bent 30° ventrad, apex expanded slightly, ventral apex with patch of ~ 10 variable-length short spikes, dorsal apex crenulated. Vesica ~ 2.5 × phallus length, widened slightly at base, then tubular, coiled counter-clockwise slightly over 720°. *Female genitalia*: Papilla analis base dorsoventrally asymmetrical, ventral aspect extending proximal to dorsolateral attachment of apophysis, lateral base bulging laterally, distal ⅔ of papilla tapered evenly to rounded point, ventral surface and entire apex with multiple similar-length hair-like setae. Segment A8 length 1 × width, glabrous. Posterior apophysis 1.75 × segment A8; anterior apophysis 0.7 × posterior apophysis. Ostium bursae lightly sclerotized, funnel-shaped, slightly wider than posterior ductus bursae. Ductus bursae straight, length 1.5 × segment A8, posterior ½ leathery, 2 × width of anterior ½, tapered from ostium bursae to mid-ductus, anterior ½ sclerotized, tubular. Corpus bursae anterior and ventral to ductus bursae, membranous, globose, length and width 1.2 × ductus bursae length; appendix bursae directed ventrad from ductus bursae origin, moderately sclerotized, smooth, coiled 360° clockwise, coil strongest at base, ductus seminalis at tip.

######## Etymology.

The species name is from Latin lacteus, meaning of milk, and mellis, honey. Milk and honey are suggested by the colors of the moth.

######## Distribution and ecology.

*Hypotrixlactomellis* (Fig. 49) occurs in central eastern Arizona and adjacent New Mexico in the United States, with the majority of examined specimens from Arizona near the Little Colorado River. A single specimen at the CNC from the Sierra Madre Oriental in northeastern Mexico indicates that it is much more widespread than suggested by the United States records. It is a seldom-collected moth but can be common when encountered.

The flight period is late July. The early stages are unknown. Like other species in the genus, *H.lactomellis* is nocturnal and comes to light.

######## Discussion.

The bicolored olive and white habitus of this moth is unusual. Although *Hypotrix* is highly diverse in color and pattern, illustrated in Lafontaine et al. (2010), this species is unlike any other in the genus. Although eye-catching in a museum specimen, the color and pattern might be cryptic against a plant with lacy silver green foliage, such as sage (*Artemisia* spp., Asteraceae).

**Figures 48–49.**
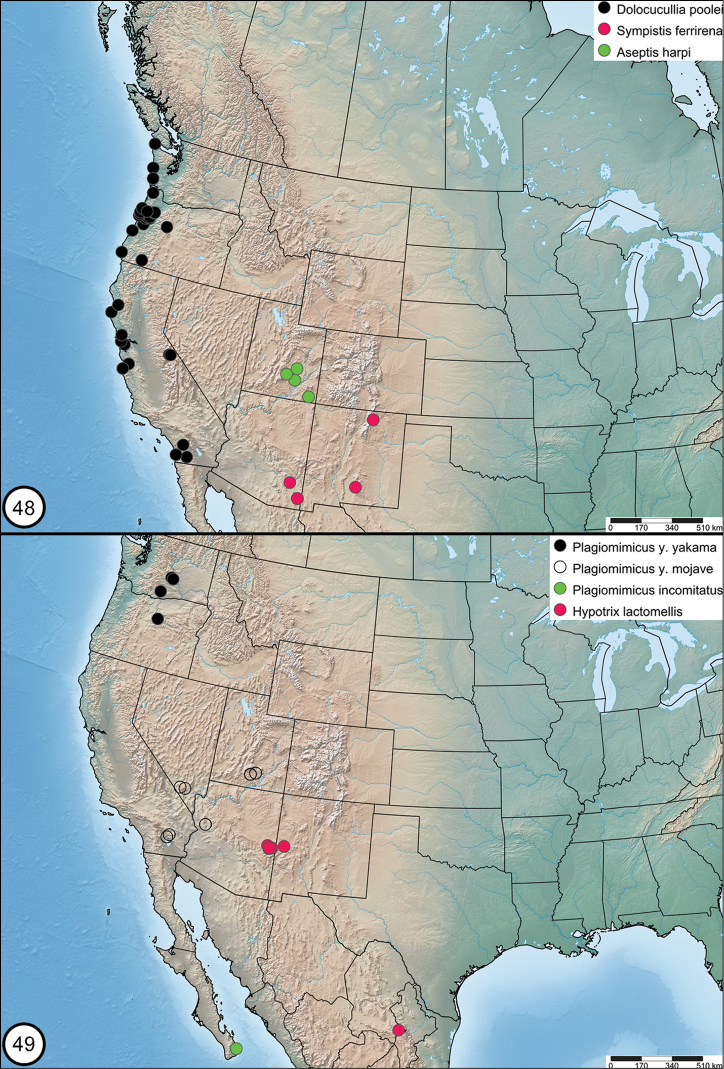
**48** Map of western North America showing distribution of examined material. *Dolocuculliapoolei* (black), *Sympistisferrirena* (red), *Aseptisharpi* (green) **49** Map of western North America showing distribution of examined material. *Plagiomimicusy.yakama* (black dot), *Plagiomimicusyakamamojave* (black circle), *Plagiomimicusincomitatus* (green), *Hypotrixlactomellis* (red).

## Supplementary Material

XML Treatment for Dolocucullia poolei

XML Treatment for Plagiomimicus yakama

XML Treatment for Plagiomimicus yakama yakama

XML Treatment for Plagiomimicus yakama mojave

XML Treatment for Plagiomimicus incomitatus

XML Treatment for Sympistis ferrirena

XML Treatment for Aseptis harpi

XML Treatment for Hypotrix lactomellis

## References

[B1] Barnes W (1904) New species of North American Lepidoptera Canadian Entomologist 36: 165–173, 197–204, 237–244, 264–268. 10.4039/Ent36264-9

[B2] Barnes W McDunnough J (1913) New N. Am. Lepidoptera with notes on described species. Contributions to the Natural History of the Lepidoptera of North America 2: 93–162.

[B3] Barnes W McDunnough J (1918) Notes and new species. Contributions to the Natural History of the Lepidoptera of North America 4: 61–208.

[B4] Grote AR (1886) On Plagiomimicusrichii. Canadian Entomologist 18: 99–100. 10.4039/Ent1899-5

[B5] Hardwick DF (1950) Preparation of slide mounts of lepidopterous genitalia. The Canadian Entomologist 10: 231–235. 10.4039/Ent82231-11

[B6] Hebert PDN Cywindka A Ball SL deWaard JR (2003) Biological identifications through DNA barcodes. Proceedings of the Royal Society of London. Series B. Biological Sciences 270: 313–321. 10.1098/rspb.2002.2218 12614582 PMC1691236

[B7] Hodges RW Dominick T Davis DR Ferguson DC Franclemont JG Munroe EG Powell JA (1983) Check List of the Lepidoptera of America North of Mexico. E. W. Classey and The Wedge Entomological Research Foundation, Washington DC, 284 pp.

[B8] Lafontaine JD (2004) The Moths of North America including Greenland, Fascicle 27. 1, NoctuoideaNoctuidae (part) Noctuinae (part—Agrotini). The Wedge Entomological Research Foundation. Washington DC, 385 pp.

[B9] Lafontaine JD Ferris CD Walsh JB (2010) A revision of the genus *Hypotrix* Guenée in North America with descriptions of four new species and a new genus (Lepidoptera, Noctuidae, Noctuinae, Eriopygini). Zookeys 39: 225–253. 10.3897/zookeys.39.438 PMC4109474 25061383

[B10] Morrison HK (1875) Notes on the Noctuidae, with descriptions of certain new species. Proceedings of the Academy of Natural Sciences of Philadelphia 1875: 55–71.

[B11] Mustelin T Crabo LG (2015) Revision of the genus *Aseptis* McDunnough (Lepidoptera, Noctuidae, Noctuinae, Xylenini) with a description of two new genera, *Paraseptis* and *Viridiseptis*. ZooKeys 527: 57–102. 10.3897/zookeys.527.9575 PMC4668888 26692788

[B12] Poole RW (1995) Noctuoidea, Noctuidae (part), Cucullinae, Stiriinae, Psaphidinae (part). In: Dominick RB, et al. (Eds) The Moths of America North of Mexico. Fascicle 26.1. The Wedge Entomological Research Foundation, Washington, DC, 249 pp.

[B13] Ratnasingham S Hebert PDN (2013) A DNA-based registry for all animal species: The Barcode Index Number (BIN) System. PLoS ONE 8: e66213. 10.1371/journal.pone.0066213 PMC3704603 23861743

[B14] Seitz A (1924) Gross-Schmetterlinge der Erde. Abteilung II. Amerikanischen Faunengebieter. Band 7. Eulenartige Nachtfalter. Alfred Kernen, Stuttgart, 508 pp.

[B15] Smith JB (1899) New noctuids and notes. Journal of the New York Entomological Society 7: 223–234.

[B16] Smith JB (1908) New species and genera of the Lepidopterous family Noctuidae for 1907 (Part II). Annals of the New York Academy of Sciences 18: 91–127. 10.1111/j.1749-6632.1908.tb55098.x

[B17] Troubridge JT (2008) A generic realignment of the Oncocnemidini*sensu* Hodges (1983) (Lepidoptera: Noctuidae: Oncocnemidinae), with descriptions of a new genus and 50 new species. Zootaxa 1903: 1–95.

